# Advances in Camptothecin-Class Compounds Nanomedicines: A Comprehensive Review of Antitumor Strategies

**DOI:** 10.3390/pharmaceutics18060712

**Published:** 2026-06-10

**Authors:** Mingliang Su, Zhiwei Liang, Long Chen, Taoyu Wang, Shabatula Aisika, Yanbin Peng, Yonghui Wu, Huahe Zhu, Lixin Wang

**Affiliations:** 1 College of Clinical Medicine, Gansu University of Chinese Medicine, Lanzhou 730101, China; myuong@foxmail.com; 2 Department of Integrated Traditional Chinese and Western Medicine, Shanghai Pulmonary Hospital Affiliated to Tongji University, Shanghai 200433, China; frank020829@163.com (Z.L.);; 3 School of Medicine, Tongji University, Shanghai 200092, China; 4 Yueyang Hospital of Integrated Traditional Chinese and Western Medicine, Shanghai University of Traditional Chinese Medicine, Shanghai 200437, China

**Keywords:** camptothecin-class compounds, tumor treatment, nanomedicine delivery, targeted delivery, tumor microenvironment response

## Abstract

Camptothecin-class compounds are anticancer active ingredients extracted from the Chinese unique medicinal plant, Camptotheca acuminata. They have been widely applied in the treatment of various cancers due to their high efficacy and broad-spectrum anti-cancer properties. This article summarizes the latest research progress over the past decade on various types of nanocarriers for camptothecin drugs. We discuss the nanodrug delivery systems for camptothecin compounds in four perspectives: passive targeting nanoparticles, active targeting nanoparticles, tumor microenvironment-responsive nanoparticles and exogenous stimulus-responsive nanoparticles. We also elaborate on the advantages of delivery nanoparticles, in vivo release characteristics and antitumor therapeutic effects. The purpose of this article is to provide a theoretical basis and innovative perspectives for the clinical application of the camptothecin drugs and the development of new pharmaceuticals.

## 1. Introduction

**Figure 1:**
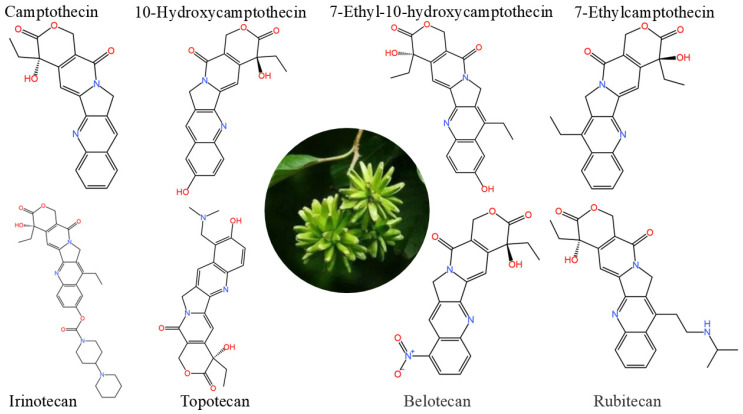
The chemical forms of CPT and its derivatives approved as chemotherapeutic agents.

Cancer is the leading cause of death worldwide and one of the most serious threats to human health and life. According to the data from the 2022 Global Cancer Report released by The International Agency for Research on Cancer (IARC), approximately 20 million new cases and nearly 9.7 million cancer deaths globally [1]. In China, statistics indicate that there were 4,824,700 new cancer cases and 2,574,200 new cancer-related deaths in 2022. Confirming cancer is still a major public health issue [2]. Traditional chemotherapy, due to the non-specific distribution of standard drugs throughout the body, affects both cancer cells and healthy cells, leading to severe toxic side effects and suboptimal treatment outcomes. Traditional Chinese medicine (TCM) has the effect of promoting tumor bearing survival, preventing recurrence, preventing metastasis and improving the quality of patient’s life. Research has indicated that certain Chinese medicine monomers could cause mitochondrial dysfunction of tumor cells, block cell cycle, inhibit proliferation, migration and invasion, promote apoptosis of tumor vascular endothelial cells, inhibit angiogenesis, etc. [3]. Camptothecin (CPT) is an indole alkaloid extracted from Camptotheca acuminata, which is a native Chinese herbal medicine. It is a topoisomerase I inhibitor and exerts natural broad-spectrum antitumor activity including colorectal, pancreatic, ovarian, lung, gastric, liver, breast, cervical, hematological cancers, and gliomas [4]. Subsequently, over 20 individual compounds were isolated from Camptotheca, demonstrating antitumor activity across multiple cancers through antiproliferative and apoptosis-inducing effects on tumor cells [5,6]. However, camptothecin-derived monomers suffer from poor water solubility, unstable lactone ring structure, short half-life, and easy adverse reactions, limiting their clinical application [7]. Camptothecin itself has never been approved for clinical use. To overcome these drawbacks, extensive chemical modification efforts have yielded numerous semisynthetic derivatives. The chemical structures of these camptothecin derivatives are displayed in Figure 1. And their detailed physicochemical properties, therapeutic efficacy, and toxic side effects are summarized in Table 1. Through structural modification of at the C7, C9, C10 and C11 positions of camptothecin ring system to improve water solubility, stability and antitumor activity, scientists developed irinotecan, topotecan (TPT), belotecan, 10-hydroxycamptothecin (HCPT) with better antitumor activity and lower toxic side effects. The development and commercialization of these drugs have played a significant role in the clinical management of tumors such as colorectal cancer, ovarian cancer, and pancreatic cancer. Nevertheless, they still cause numerous adverse toxic effects, including myelosuppression and delayed-onset diarrhea. Consequently, there is an urgent need to develop and optimize nano-dosage forms of camptothecin-based anticancer agents. Nanoparticle delivery systems represent a promising strategy for camptothecin and its derivatives. By encapsulating these agents, nanocarriers improve their aqueous solubility and in vivo bioavailability. Meanwhile, the unstable lactone moiety can be shielded from early hydrolysis within the delivery vehicles. Such systems also enable tumor-selective accumulation through the enhanced permeability and retention (EPR) effect or specific ligand–receptor interactions. Furthermore, controlled or stimuli-triggered drug release minimizes systemic adverse effects while elevating local drug levels inside tumors.

**Figure 2:**
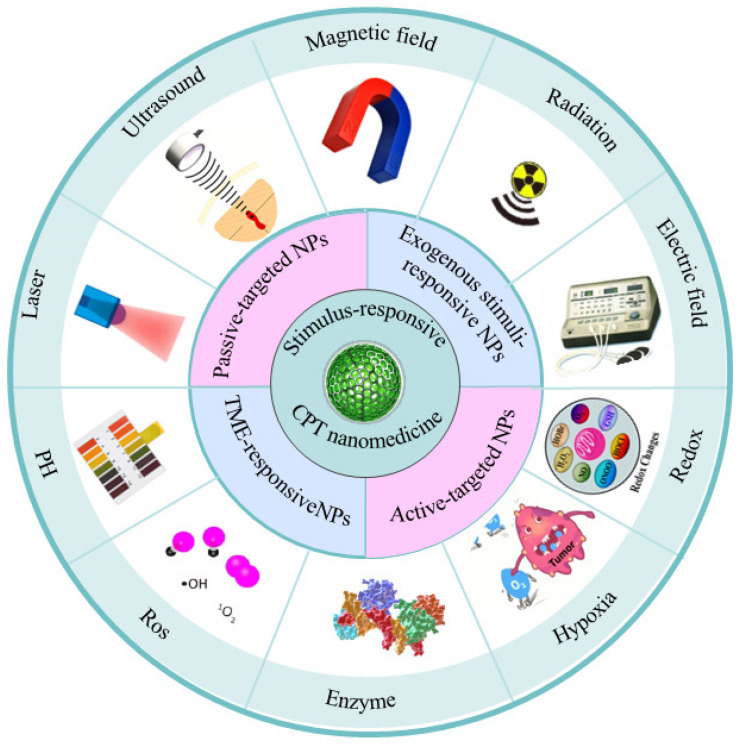
Schematic diagram of CPT-class compound nanomedicine delivery systems and their stimulus-responsive types.

In 1965, the British scientist Bangham firstly proposed the concept of nanolipid vesicles [8]. Since then, numerous nanodrug delivery systems such as liposomes, polymeric nanoparticles and micelles have been developed for cancer treatment. These formulations can improve drug stability and solubility, facilitate transmembrane transport, extend in vivo circulation time, inhibit drug degradation, realize active targeting, lower administration dosage and alleviate systemic adverse reactions [9,10,11,12]. Over the past few decades, nanotechnology-based camptothecin preparations have made great strides in cancer therapy, which has overcome the problems of poor water solubility, poor targeting and severe toxic side effects of traditional camptothecin preparations [13,14,15,16]. However, camptothecin nanopreparations still encounter difficulties in clinical transformation, such as insufficient accumulation and penetration efficiency in tumors. Therefore, it is necessary to comprehensively summarize the recent research progress of camptothecin-based nanomedicines. Although several previous reviews have reported camptothecin-related delivery systems, most of them only focus on specific carrier types or single therapeutic applications, and few studies have established a systematic classification framework that integrates material properties, targeting mechanisms and stimulus-responsive characteristics. This review systematically sorts out the domestic and foreign research progress of camptothecin nanoparticles in the past decade. Different from previously published reviews, this paper classifies camptothecin nanocarriers into four categories based on their material characteristics, targeting modes and stimulus-responsive behaviors, including passively targeted nanoparticles, actively targeted nanoparticles, tumor microenvironment-responsive nanoparticles, and exogenous stimulus-responsive nanoparticles (Figure 2). This article further thoroughly investigates the current status of camptothecin-based nano-delivery systems and elaborates their unique advantages, in vivo release behaviors and antitumor effects. In addition, we summarize and analyze how different nanoformulations resolve the inherent defects of camptothecin compounds, such as poor water solubility, unstable lactone ring structure and serious systemic toxicity, and compare the therapeutic characteristics of different nanoplatforms. This paper also discusses the applicability of various formulations in different tumor types and clinical scenarios, so as to provide references for the rational structural design of camptothecin nano-preparations. This review aims to provide empirical support for the further development of novel camptothecin compounds nanoparticles and promote the clinical transformation of such antitumor nanomedicines.

## 2. Passive Targeted Nanodrugs

**Figure 3:**
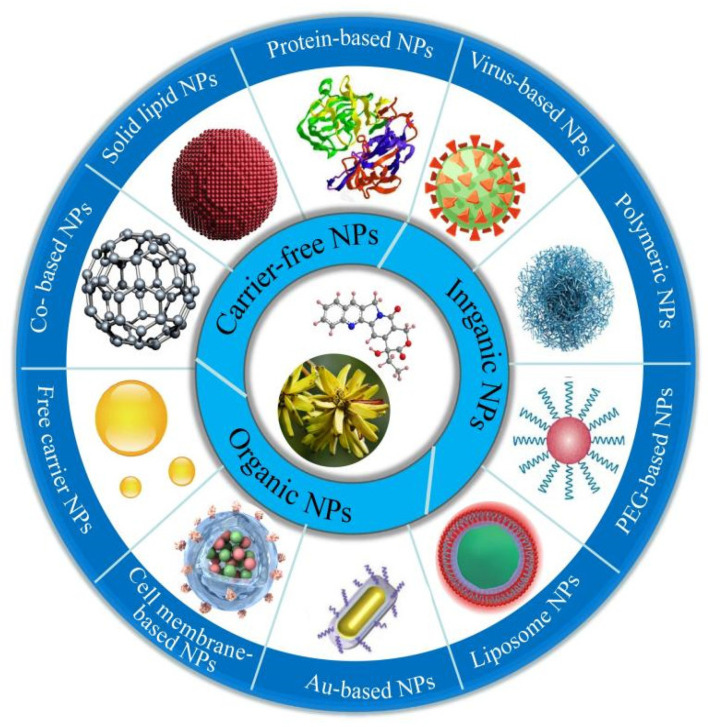
Schematic diagram of the composition classification of CPT-class compounds nanomedicine delivery carriers.

Compared with conventional drugs, nanoparticles (NPs) can deliver drugs to tumor tissue, maintain drug release and efficiently treat lesions over a long period of time, offering a promising approach to improve therapeutic accuracy and reduce toxic side effects on healthy tissues [17]. Passive targeting is realized by associating drugs with carriers of specific particle size and surface characteristics, through phagocytosis, adsorption or exchange of target organs or cells [18], whereas EPR effects of tumor tissues further facilitate the accumulation of nanodrugs at tumor sites [19]. Main camptothecin NPs include various types of drug delivery systems like liposomes, polymer micelles, nanoparticles, microspheres, polymer precursors, etc. (Figure 3).

### 2.1. Lipidosome

**Figure 4:**
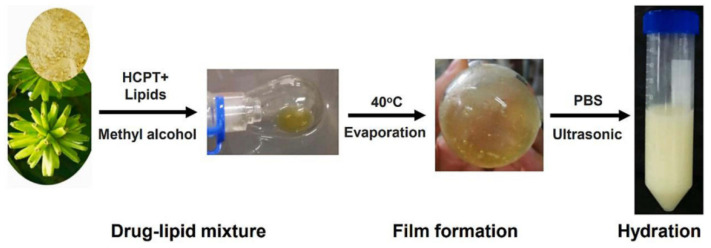
Schematic diagram of the preparation of HCPT liposomes. Reproduced with permission [22]. Copyright 2021, Elsevier.

Nanoliposome technique was first conceived by AlecD Bangham in the 1960s. Lipid nanomaterials have a membrane structure that is highly analogous to that of cell membranes. It can channel effective components into cells after entrapping active ingredients to facilitate their cellular entry. Camptothecin lactone ring structure is unstable, susceptible to decomposition under light; Encapsulating camptothecin-based drugs within liposomes enhances their structural stability [20]. Chen Wenzhong [21] prepared hydroxycamptothecin liposomes by thin film dispersion method with entrapment efficiency higher than 93%. This approach protects the active δ-lactone ring structure of hydroxycamptothecin. Compared to commercially available hydroxycamptothecin injections at equivalent doses. At the same dosage, hydroxycamptothecin liposomes exerted more pronounced inhibition against mouse transplanted tumors H22 liver cancer and S180 sarcoma than commercialized hydroxycamptothecin injection while reduced the toxicity of hydroxycamptothecin. Xiao et al. [22] prepared cationic HCPT liposomes by the thin film method, and combined with 5-aminolevulinic acid (5-ALA) intratracheal injection for chemotherapy and sonodynamic therapy (SDT) of metastatic lung cancer (Figure 4). This nanodrug exhibits high anticancer efficacy in metastatic lung cancer-bearing mice. Chemo-SDT group exhibited fewer tumor nodules and reduced infiltration of cancer and inflammatory cells relative to the model group. In particular, this synergistic treatment achieved superior therapeutic outcomes, with negligible residual tumor lesions and intact alveolar structures.

### 2.2. Polymer Nanomedicine

Polymeric nanoparticles are drug delivery systems that encapsulate drugs within natural or synthetic polymeric carriers, commonly appearing as nanoparticles or micelles. Nanoparticles and micelles are typical representatives. Compared with liposomes, polymer materials are extensively applied in the preparation of nanoparticles for their higher drug loading, better circulation, biodegradability and controllable release [23]. Polymer nanoparticles can be classified into inorganic polymer nanoparticles and organic polymer nanoparticles based on the composition and structure of polymers. Inorganic nanoparticles possess simple preparation methods, rich constituent elements and good stability while organic nanoparticles exhibit better biocompatibility.

Organic NPs encompassing polyethylene glycol (PEG), poly lactic acid-co-glycolic acid (PLGA), polylactic acid (PLA), lipids, gels, dendrimers, cyclodextrins, electrospun fibers, polysaccharides, etc., possess high cellular uptake, biodegradability and loading efficiency. Han Lu et al. [24] grafted PEG monomethyl ether (mPEG) with 10-HCPT by utilizing aconitic anhydride (CA) as a linker to form amphiphilic polymers. The nanometer material improves drug loading efficiency, mPEG modification can reduce liver deposition, enhance tumor passive targeting via EPR effect and improve anticancer efficacy. Qiao et al. [25] linked 10-HCPT to polyhydroxy poly(malic acid) through glycine to generate a poly malic acid–hydroxycamptothecin conjugate. This conjugate ameliorates the water solubility of 10-HCPT. It can release active HCPT through ester bond cleavage in the tumor microenvironment, thereby producing potent antitumor effects. Nano-gels, with their small particle size, high solubility, and drug-loading capacity, serve as ideal carriers for camptothecin-based drugs. Particularly, positive nanogels can efficiently load camptothecin, maintain its stability, and enhance in vivo retention and tumor tissue permeability, thus greatly improving anticancer effects [26]. Lu et al. [27] encapsulated SN-38 (7-ethyl-10-hydroxycamptothecin) into micelles self-assembled from mPEG-PLA; this approach not only enhanced drug targeting, but also prolonged the retention time of drugs in vivo, enabling it could play a sustained release role in tumor sites. In vivo experiments have demonstrated that SN38-conjugated micelles exhibit reduced systemic toxicity and enhanced antitumor efficacy. Liu et al. [28] stably embedded a cleavable camptothecin precursor drug in methoxypolyethylene glycol-polylactic acid (mPEG-PLA) micelles. This system can respond to tumor microenvironment to release drugs, with excellent tumor targeting ability and high potential for camptothecin drug delivery.

**Figure 5:**
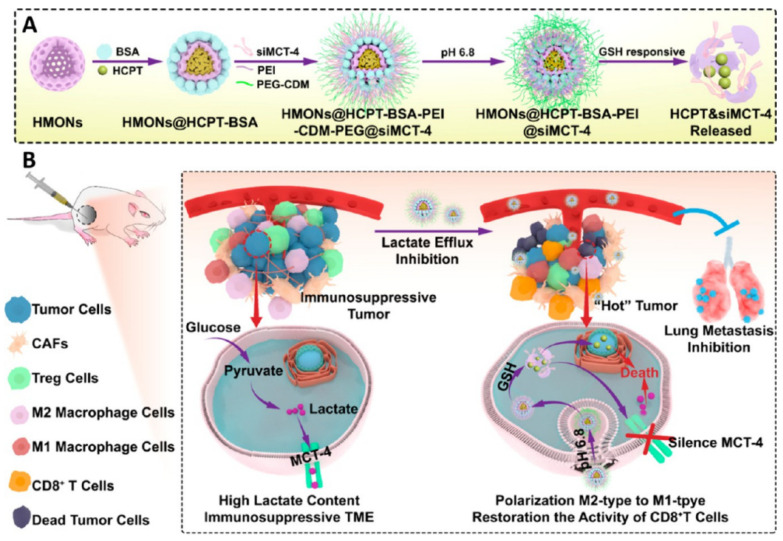
Schematic illustration of the cascaded responsive nanoplatform for enhancing tumor chemo-immunotherapy via inhibiting lactic acid efflux. (**A**) Synthesis of HMONs@HCPT-BSA-PEI-CDM-PEG@siMCT-4 and stimuli-responsive degradation. (**B**) The nanoplatform directly induces tumor cell apoptosis though HCPT and increased intracellular lactate, then transforms immunosuppressive tumors to “hot” tumors, polarizes the TAM phenotype from M2 type to M1 type, and restores CD8+ T cell activity via inhibiting lactate efflux [30]. Copyright 2020, American Chemical Society.

Compared with organic nanoparticles, inorganic nanoparticles such as silica (SiO_2_), gold (Au), magnetic nanomaterials, graphene oxide (GO), copper (Cu), Prussian blue, platinum (Pt) and calcium carbonate (CaCO_3_) exhibit stable structure and great potential for cancer therapy [29]. Li developed a smart controlled-release nanopharmaceutical responsive to tumor microenvironment by using hollow mesoporous silica nanoparticles (SiO_2_ NPs) as carriers, simultaneously loaded with HCPT and siMCT-4 (Figure 5). The nanoplatform effectively improved the immunosuppressed tumor microenvironment by inhibiting lactic acid efflux combined with chemotherapy, this nanoplatform effectively improved the immunosuppressive tumor microenvironment by inhibiting lactic acid efflux in combination with chemotherapy, significantly suppressing tumor growth and reducing lung metastasis in B16F10 melanoma cells and 4T1 breast cancer cells [30]. Li et al. successfully prepared Au NPs loaded with HCPT by electrostatic deposition method; these nanoparticles exhibit chemical inertness, ease of synthesis, size controllability, and strong surface modifiability, enabling controlled drug release under near-infrared (NIR) light irradiation [31]. In vitro and in vivo findings collectively validate the prominent synergistic antitumor efficacy of combined localized chemotherapy and NIR-mediated photothermal therapy (PTT), which outperforms single-treatment modalities. More importantly, due to the high drug-loading content and efficient photothermal effects of the nanocomposites, 100% in vivo tumor elimination is achieved at a low laser irradiation power density of 1 W cm^−2^ without weight loss and tumor recurrence. Moreover, Bao et al. further investigated the antitumor effect of Au NPs of different sizes loaded with HCPT. In vivo studies on MDA-MB-231 breast cancer xenografts demonstrated that the antitumor efficacy of HCPT-AuNPs was highly size-dependent. Au NPs with average diameter of about 50 nm showed the strongest inhibitory effect on mouse MDA-MB-231 tumor, significantly outperforming free HCPT as well as 10 nm and 25 nm counterparts, with the latter two showing no obvious efficacy difference [32].

Carbon nanotubes (CNTs) are tubular structures composed of carbon atoms, serving as highly effective drug delivery carriers with a high drug loading capacity. Due to their large specific surface area, drug molecules can be attached to their surfaces or encapsulated in their interior. Sahoo et al. [33] loaded camptothecin onto polyvinyl alcohol (PVA) functionalized multi-walled carbon nanotubes (MWCNTs) and GO with high hydrophilicity and biocompatibility by using non-covalent supramolecular attachment technique. Compared to free camptothecin, MWCNT-PVA-CPT and GO-PVA-CPT exhibited significantly enhanced antitumor activity against MDA-MB-231 human breast cancer cell line, and their potency was increased by about approximately 15-fold.

### 2.3. Albumin-Type Nanoparticles

As the most abundant, highly concentrated, and long-circulating protein in blood, albumin serves not only as a therapeutic agent but also as a drug carrier [34]. More interestingly, the albumin type nanoparticles can escape the attack of the immune system due to their non-immunogenicity, and can also prolong the circulation half-life of drugs and facilitate the accumulation of drugs in tumors [35]. They are suitable for encapsulating camptothecin drugs with a short half-life in vivo. Yang Zhenbo [36] prepared 10-hydroxycamptothecin human albumin nanoparticles (HCPT-HSA-NPs) based on low molecular weight PEG and 10-hydroxycamptothecin liquid drug complex (l-PEG-HCPT). HCPT-HSA-NPs achieved over 99% encapsulation efficiency, demonstrated excellent solution stability, and exhibited sustained-release capabilities. In vitro experiments verified that HCPT-HSA-NPs exert stronger tumor cell cytotoxicity than free HCPT injection. In vivo, this nanoformulation enables sustained drug release within tumor tissues, prolonging local drug exposure and thereby improving antitumor efficacy. Overall, HCPT-HSA-NPs exhibit the most prominent anticancer activity among all treatment groups. Zu et al. [37] constructed glycyrrhizic acid–bovine serum albumin (GL-BSA) nanoparticles loaded with hydroxycamptothecin (HCPT), abbreviated GL-BSA-HCPT-NPs. An in vitro release study showed that compared with free HCPT, this nanomedicine could achieve high sustained release effect. Its IC_50_ value was significantly lower than that of free HCPT after incubated with SMMC7721 human hepatoma cells, demonstrating better anticancer activity.

### 2.4. Drug Crystalline Nanoparticles

Drug crystal nanoparticles are nano-sized drug crystals made by nano-technology. Because of its ultra-small particle size, it has a very large specific surface area, which can improve the solubility and dissolution of insoluble drugs, improve their bioavailability, and improve their in vivo distribution [38,39]. Wang et al. [40] successfully prepared HA-coated camptothecin nanocrystals via surface functionalization strategy. Nanometer crystal has higher drug loading efficiency, can extend the in vivo circulation time, enhance the stability of camptothecin, increases the selective uptake of drugs by targeting delivery of HA, and significantly enhances the antitumor activity and cancer cell apoptosis induction ability of CPT. Chen et al. developed carrier-free HCPT and DOX assembled positively charged microparticles composite nanoparticles, this positive charge helped to enhance the cellular uptake of HCPT nanoparticles. This positive charge facilitates cellular uptake of HCPT nanoparticles, significantly improving bioavailability compared to free HCPT [41]. Han self-assembled and synthesized carrier-free HCPT nanosuspension with uniform size and good dispersibility by precipitation–ultrasonic treatment method, enhancing the solubility of drugs. This nanopreparation exhibited stronger anticancer activity than conventional HCPT injection in H22 tumor mouse model and alleviated side effects [42].

### 2.5. Nucleic Acid Nanoparticles

**Figure 6:**
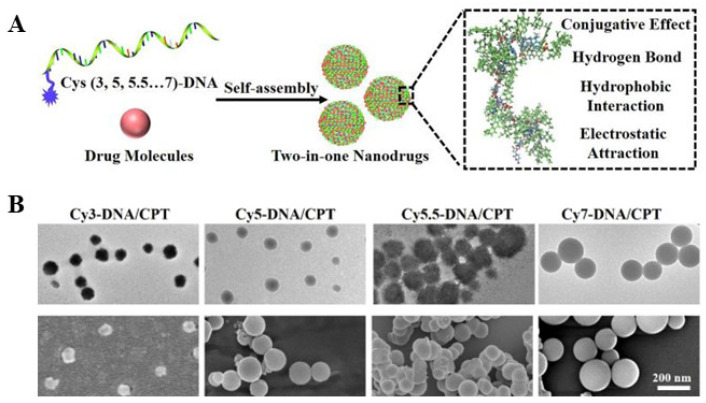
(**A**) Schematic illustration of the self-assembly process of the two-in-one nanomedicine. (**B**) Transmission electron microscopy (TEM) and scanning electron microscopy (SEM) images of Cy3, 5, 5.5, 7-DNA/CPT nanospheres. Reproduced with permission [45]. Copyright 2021, Wiley-VCH.

DNA nanotechnology takes advantage of the programmability of nucleic acids to generate self-assembled structures that hold significant promise for biological applications such as imaging, sensing, and drug delivery [43]. Zhang J et al. [44] built a DNA nanomedicine tetrahedral skeleton via carbethylbromine-modified camptothecin (CPT) and phosphorothioate (PS)-modified DNA self-assembly. The water solubility and base pairing ability of DNA were maintained by regulating the number and location of CPT. CPT-TET has higher cellular uptake and cytotoxicity to tumor cells in vitro. In a tumor-bearing mouse model, CPT-TET shows enhanced accumulation at the tumor site owing to its nanosized feature. During the treatment, CPT-TET exhibited a more potent therapeutic efficacy than control groups and dramatically delayed the tumor progression. All these results demonstrate that CPT-TET can elicit impressive therapeutic efficacy without the severely side effects. Han Xiao et al. [45] designed a universal self-assembly platform, directly interacting with various chemotherapeutic drugs by cysteine-modified DNA (Cys-DNA) such as CPT, without additional carriers. Cys-DNA strengthened the non-covalent interaction with CPT, spontaneously formed multifunctional hybrid nanospheres, realizing efficient drug loading and accurate control (Figure 6). This nanomedicine platform demonstrates broad potential for combined chemotherapy and gene therapy in antitumor applications.

### 2.6. Porous Crystalline Nanocarriers

**Figure 7:**
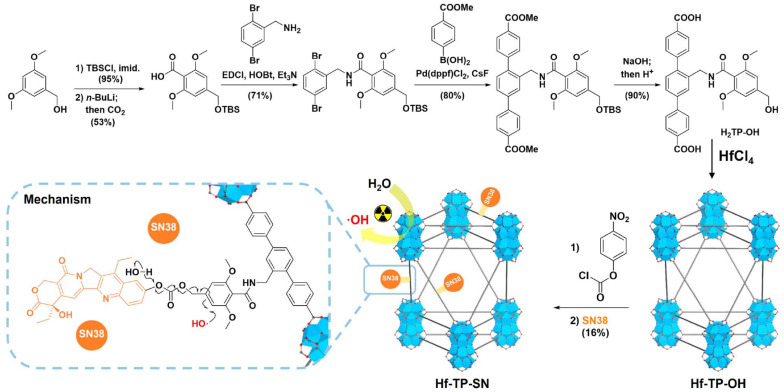
Synthesis of Hf-TP-OH nMOF and its postsynthetic modification with SN38 to afford Hf-TP-SN nMOF along with the proposed mechanism for X-ray triggered release of SN38 from Hf-TP-SN. Reproduced with permission [46] Copyright 2023, American Chemical Society.

In addition, other types of camptothecin nanodrug delivery systems, including metal–organic frameworks (e.g., ZIF-8, Fe-MOF) and covalent organic frameworks (COFs), have also been reported to have enhanced antitumor effects and potential clinical applications. Porous nanocarriers possess large specific surface areas, tunable pore structures and versatile surface modification sites. These structural advantages enable both high-efficiency drug loading and intelligent delivery. Lin Wenbin’s team [46] constructed an X ray-responsive nMOF (Hf-TP-SN) covalently conjugated to the SN38 prodrug (Figure 7). The Hf_12_ units within it function as radiosensitizers, boosting hydroxyl radical (·OH) production and triggering a cascade reaction in the 3,5-dimethoxybenzyl carbonate. This mechanism results in fivefold more SN38 release compared to molecular prodrugs. In experiments, Hf-TP-SN combined with X-ray irradiation exhibits significant cytotoxicity against cancer cells and effectively inhibits tumor growth in mouse models of colon and breast cancer. While PBS(+) moderately inhibited the growth of CT26 and 4T1 tumors with tumor growth inhibition indices (TGIs) of 0.468 and 0.328, respectively, Hf-TP-SN(+) potently inhibited CT26 and 4T1 tumors with TGIs of 0.965 and 0.889, respectively. Notably, the Hf-TP-SN (+) formulation could completely eliminate CT26 tumors in 40% of treated mice. Such superior in vivo therapeutic performance is attributed to the synergistic effects of nMOF-based radiosensitization and X-ray-triggered SN38 drug release of Hf-TP-SN nanocomposites.

A pH-responsive and mitochondria-targeted liposome-enveloped methoxylated covalent organic framework (mCOF) delivery system loaded with doxorubicin (DOX) and camptothecin (CPT) is constructed [47]. Cholesteryl hemisuccinate is assembled with amphiphilic DOX-lipid on the surface of the mCOF to achieve pH-sensitive drug release, mitochondrial targeting, and therapeutic agent delivery (DOX) to induce oxidative stress in tumor mitochondria. Furthermore, CPT, which can also induce mitochondrial oxidative stress, is co-loaded with DOX-lipid using mCOF with high porosity to enhance the synergistic therapeutic effects. In addition, this combination promoted synergistic amplification of mitochondrial dysfunction and significantly enhanced ROS generation. CPT@mCOF@DOX-lipid exhibited superior tumor-suppressive efficacy over other treatment groups in vivo, which stemmed from its mitochondrial targeting capability and the synergistic antitumor effect of CPT and DOX dual drugs. The results further validated that the integrated advantages of pH-responsive drug release, mitochondria-targeted precise delivery, and synergistically elevated ROS production collectively endow this dual-delivery system with prominently enhanced tumor inhibitory performance. Consequently, the DOX-CPT dual drug-loaded delivery system has strong antitumor activity, highlighting a promising approach for organelle-specific combination chemotherapy.

Passive targeting nanomedicines can enrich drugs in tumor tissues via EPR effect. Although they can enhance drug efficacy and reduce toxic and side effects, their targeting efficiency is restricted because the amount of drug accumulation in tumor sites is limited by the microenvironment structure. In order to further strengthen drug targeting and therapeutic effects, researchers have developed nanoparticles with active targeting functions to realize accurate drug delivery.

## 3. Active Targeted Nanodrugs

Precision therapy has been a focal point in pharmaceutical research for a considerable period of time. Active targeting nanoparticles have been demonstrated to exhibit superior targeting properties in comparison to passive targeting nanoparticles. Active targeting generally involves utilizing specially modified drug carriers as missiles to attach targeted ligands or antibodies onto the surface of NPs through the ligand–receptor recognition mode to allow for recognition by target tissues or cells, imparting to drugs the capacity to actively bind to targets, specifically delivering therapeutic drugs to the sites at which they are to exert their effects, and enhancing efficacy whilst decreasing adverse effects [48].

### 3.1. Polypeptide–Antibody Drug Conjugate Nanoparticles

**Figure 8:**
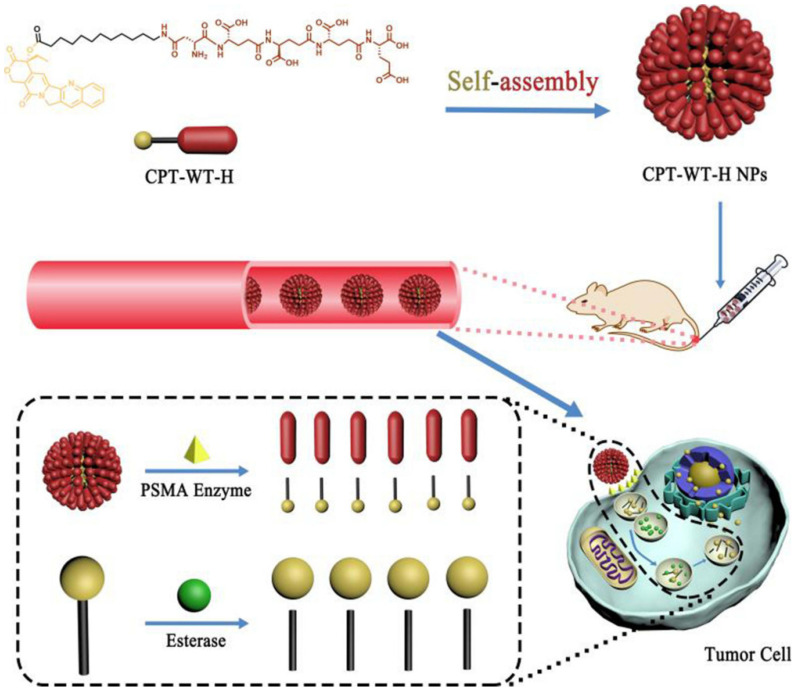
Schematic diagram of the synthesis and antitumor mechanism of PSMA and esterase dual-responsive CPT nanoparticles (CPT-WT-H NPs). Reproduced with permission [52]. Copyright 2021, Dove Medical Press Limited.

Peptide drug conjugates (PDCs) and antibody drug conjugates (ADCs) have become the focus of research into nanodrugs due to their low immunogenicity, simple synthesis process, and good biocompatibility. PDC utilizes both natural and synthetic peptides as small ligands derived from known proteins in the body. ADC delivers drugs to cancer cells by binding to the specificity of monoclonal antibodies that can specifically bind to the receptors overexpressed by cancer cells. Compared with traditional chemotherapy, these nanomedicines can significantly enhance drug targeting, improve tumor cell permeability, and reduce side effects, demonstrating great potential for application [49,50]. Wang et al. [51] designed a PDC drug SAP-CPT assembled by targeting motif (RGD target), assembly motif (GNNNQNY) and CPT molecule. After binding with tumor overexpression receptor integrin alpha V beta 3 on the cell surface, SAP-CPT forms nanoclusters in situ, thus improving the efficiency of PDC drugs entering cells. It showed higher therapeutic effect than CPT single molecule in MCF-7 breast cancer and EJ bladder cancer xenograft mouse models, improving the maximum tolerated dose of drugs, and solving the problem of poor permeability of PDC drugs to tumor cells. Xu Bing et al. [52] was inspired by the prodrug principle to combine the hydroxyl group at position 20 of hydrophobic CPT with the hydrophilic prostate specific membrane antigen (PSMA) response pentapeptide through a cleavable ester bond to construct the tumor-targeted camptothecin prodrug CPT-WT-H NPs (Figure 8). Dual-responsive nanopharmaceuticals with negatively charged surfaces first respond to extracellular PSMA and then to cellular lactonases, realizing programmable release of CPT at tumor sites, improving CPT delivery efficiency. CPT-WT-H NPs showed good tumor targeting and significant antitumor effect in vitro and in vivo with low toxicity.

In recent years, camptothecin-based topoisomerase I inhibitors have become one of the most promising classes of ADC payloads. IMMU-132 is a humanized anti-Trop-2 antibody conjugated to SN-38 through a pH-sensitive CL2A hydrazine linker. It delivers SN-38 drugs to cancer cells accurately by targeting Trophoblast Cell-Surface Antigen 2 (Trop-2) positive metastatic triple negative breast cancer (mTNBC) and locally advanced or metastatic urothelial cancer (mUC). The linker is cleaved within the acidic environment of cancer cells, thus facilitating the controlled release of drugs and achieving a significant anticancer effect [53]. Statistics from UmabsDB [54] shows that there are now more than 80 ADC drugs under investigation in clinical phase using camptothecin derivatives as small molecule toxins, involving 24 targets, over 15 payloads and 21 linkers.

### 3.2. Small-Molecule Ligand Nanoparticles

It is an established fact that small-molecule ligands are characterized by a reduced molecular weight, a simplified synthesis process, a diminished immunogenic potential, and an enhanced capacity for penetration into tumor cells. Additionally, the smaller targeting moiety also dictates that nanoparticles do not aggregate readily in cells and will be quickly excreted from the body even if the drug is misdelivered, reducing the risk of toxicity to normal cells [55,56]. Folic acid (FA) and hyaluronic acid (HA) are commonly used small molecule ligands. Folate receptor (FR) and membrane glycoprotein Cluster of Differentiation 44 (CD44) are poorly expressed in normal tissues but highly expressed in tumor cells; therefore, they are frequently employed as targeting groups for nanovectors [57,58]. Wan Long et al. [59] prepared three-dimensional ordered macroporous carbon carriers using polystyrene microspheres as colloidal crystal templates. They then introduced carboxyl groups onto the surface of the three-dimensional ordered macroporous carbon carriers by wet oxidation. Finally, they grafted FA onto the carrier surface via amide bonds. FA modification increases the hydrophilicity of the drug-loaded system and greatly improves the dissolution rate and dissolution of hydroxycamptothecin. Lai Chunli et al. [60] constructed folate-terminated polyrotaxane–camptothecin (FA-PR-CPT) conjugate specifically targets folate-positive tumour cells. It facilitates endocytosis of the drug by cells and enhances cytotoxicity. No obvious receptor targeting is observed for folate-receptor negative cell lines. FA-PR-CPT exhibits obvious antitumor effect on S180 tumor-bearing mice.

**Figure 9:**
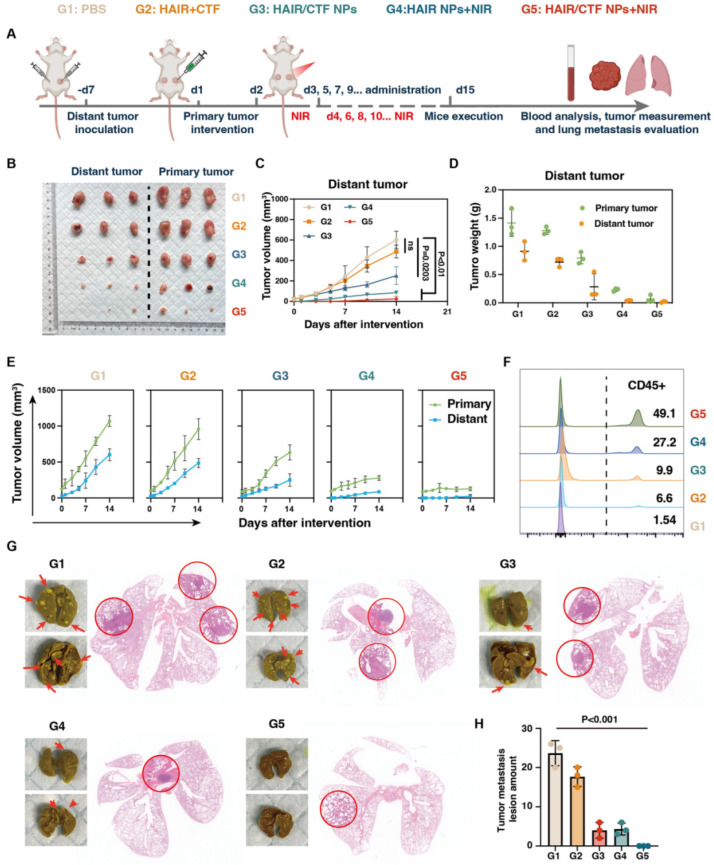
Abscopal antitumor effect of HAIR/CTF NPs. (**A**) Schematic illustration of treatments on bilateral tumor-bearing mice. (**B**) Image of primary tumor and distant tumor after different interventions. (**C**) Distant tumor volume analysis of mice in each group (*n* = 3). (**D**) Distant tumor weight of mice in each group (*n* = 3). (**E**) Bilateral tumor volume analysis of mice received diverse interventions in each group (*n* = 3). (**F**) Ratio of immune cells in mice blood after different treatments (*n* = 3). (**G**) Photographs of tumor metastasis lesion in lungs and partially enlarged (**H**,**E**) staining images after different treatments and its statistical analysis (**H**). Reproduced with permission [61]. Copyright 2024, BMC.

Inspired by the specific binding ability of HA to CD44, Xu et al. [61] developed a HAIR/CTF nanobab for triple negative breast cancer (TNBC) therapy, incorporating IR780 and modifying HAIR to co-deliver hydrophobic CPT and hydrophilic 5-fluorouracil (FUDR) and self-assemble into ROS-responsive actively targeted nanoparticles via ROS-sensitive TK linkers. HAIR/CTF can be guided to tumor site by HA. Under 808 nm laser irradiation, IR780 generates reactive oxygen species (ROS), triggers TK bond cleavage, releases CPT and FUDR, and achieves PTT and PDT synergistic killing tumor cells. Meanwhile, CPT and phototherapy induce immunogenic cell death (ICD), activate immune responses, and inhibit primary and metastatic tumors (Figure 9). HAIR/CTF NPs + NIR intervention exhibited the best tumor-killing effect whereas HAIR/CTF NPs group showed lower antitumor efficacy. Additionally, HAIR/CTF NPs + NIR group exhibited an 80% tumor cure rate whereas the HAIR NPs + NIR group only suppressed tumor growth without completely eradicated tumors. Mice that received HAIR/CTF NPs with or without NIR gained obviously longer survival rate. Consequently, the HAIR/CTF nano-bomb realized spatiotemporal controllable drug release, exciting tumor eradication and attractive anti-metastasis efficacy via combination chemo/photo/immunotherapy, offering a valuable reference for the re-development of classic drug in future clinical practice.

Alendronate (ALN), a typical nitrogen-containing bisphosphonate, possesses inherent high bone targeting capability. Owing to such specific bone-binding property, ALN is widely utilized as an efficient targeting ligand to modify nanocarriers, achieving precise bone enrichment and improving local drug retention for the treatment of bone tumors and bone-related diseases. Wang et al. [62] developed a multifunctional bone targeted delivery system of PDA-ALN with ALN as ligand and loaded with anticancer drug SN38 for targeted treatment of malignant bone tumors. The nano medicine can be released under NIR light and weak acid conditions, thus improving accumulation of the medicine in the osteolytic lesion area. Through the synergistic effects of chemotherapy and PTT, the nanomedicine can effectively control the growth of bone tumors and reduce bone destruction. It holds great potential for treating malignant bone tumors.

### 3.3. Binding Protein Nanoparticles

**Figure 10:**
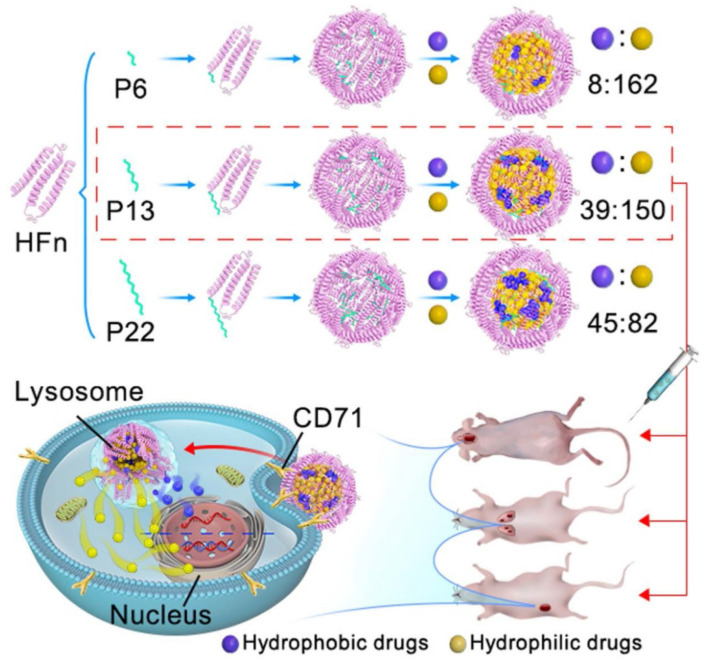
Schematic illustration of the key steps in Cpt/Epi@ins-FDC preparation, optimal PDR selection, and the synergistic antitumor activity. Reproduced with permission [64]. Copyright 2021, Ivyspring International Publisher.

The inherent targeting of ferritin by CD71 has the potential to facilitate the specific uptake of drugs by tumor cells. Furthermore, it has been demonstrated to penetrate the blood–brain barrier and efficiently accumulate in brain tumors, without the necessity for conventional nanocarrier modification [63]. Wang ZR et al. [64] designed a novel ferritin carrier ins-FDC capable of co-loading hydrophilic and hydrophobic drugs by appending hydrophobic peptide chain to C-terminal of human heavy chain ferritin single subunit, remodeling ferritin lumen and realizing co-loading of hydrophobic CPT and hydrophilic epirubicin (EPI) (Figure 10). The novel construct showed hydrophobic drug inclusion capacity, targeted drug delivery and post loading particle stability, and significant half-life extension. The drugs loaded in the inner cage of ins-FDC were detected to show the Epi-Cpt programed release.

In various mouse tumor models (glioma, metastatic liver cancer and drug-resistant breast cancer), owing to its intrinsic CD71 targeting, Cpt/Epi@ins-FDC significantly prolonged drug half-life, exhibited a higher synergistic antitumor effect than the single-drug-loaded ones and efficiently overcomes the tumor cell drug resistance. Treatment with Cpt/Epi@ins-FDC significantly prolonged animal survival. This formulation achieved a median survival of 36 days in U87MG-LUC glioma-bearing mice, 60% survival at 50 days in HepG2-LUC tumor models, and 100% survival up to 150 days in MCF7-MDR tumor-bearing mice, showing markedly superior therapeutic outcomes over free drugs and single-drug nanoformulations.

Casein (CA) is a food-derived protein with specific brain targeting. It can efficiently penetrate the blood–brain barrier (BBB) via endogenous transcytosis pathways, enabling effective brain accumulation of nanocarriers. Due to favorable characteristics, CA serves as a promising targeting ligand for constructing brain-targeted delivery systems to treat intracranial and central nervous system diseases. Gao’s study [65] shows that menthol (menthol, M, a brain barrier penetrant) was combined with CA and loaded with HCPT to form menthol-modified casein nanopharmaceuticals (HCPT-M-CA-NPs). HCPT-M-CA-NPs are highly enriched in brain tumor areas and the survival time of glioma mouse models treated with HCPT-M-CA-NPs is markedly improved, which shows certain potential in glioma therapy.

### 3.4. Biofilm-Coated Nanoparticles

When circulating in vivo, nanoparticles may be identified and cleared by the reticuloendothelial system (RES) due to their immunogenicity [66]. Research has demonstrated that the application of cell membrane coated or modified nanocarriers can overcome this issue. The membrane-coated nanoparticles demonstrate properties analogous to those of natural cell membranes. They are capable of evading immune system attack, enhancing biocompatibility and circulation time in vivo, and facilitating more efficient delivery of nanomedicines to tumor tissues via a homologous targeting effect, thereby enhancing drug bioavailability [67]. Zhang Lijun et al. [68] prepared CPT nanocrystals coated with 4T1 cell membrane and loaded with ICG (NCS-ICG-CM). Its homologous targeting enhanced drug uptake in 4T1 cells. NCS-ICG-CM prolonged drug circulation time, avoided immune cell uptake damage and enhanced tumor accumulation. Combination with phototherapy, it exhibits excellent anti-triple negative breast cancer and anti-metastasis effects and significantly prolonged survival time of mice. Lv Jingdi et al. [69] loaded HCPT onto liposomes coated with hepatoma cell membrane and modified with mitochondria targeting triphenylphosphine (TPP) positive particles to deliver HCPT efficiently to mitochondrial effect sites of hepatoma cells, gave full play to the advantage of drugs acting accurately on target organelles, induced mitochondrial dysfunction of tumor cells, thus greatly enhanced the effect of HCPT on inducing apoptosis of hepatoma cells.

**Figure 11:**
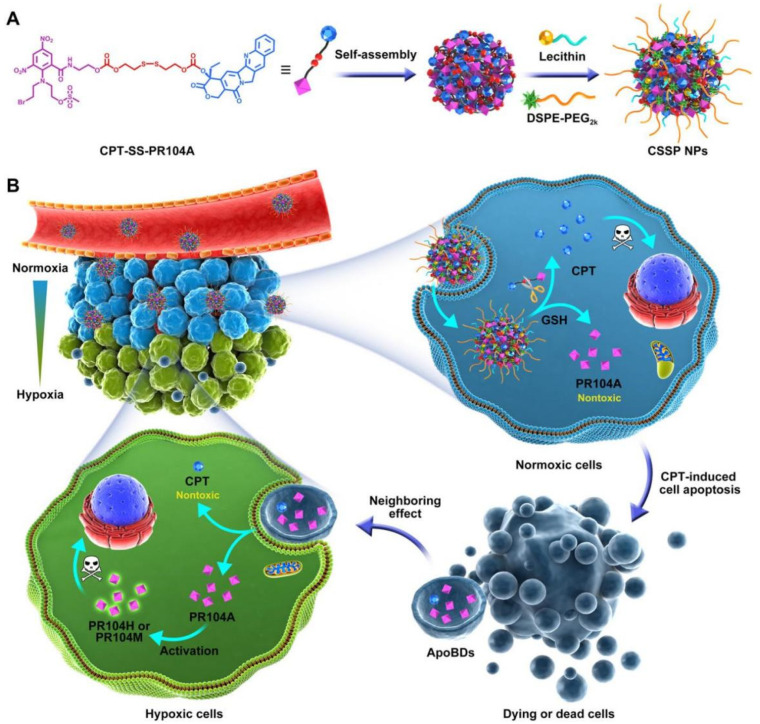
(**A**) Fabrication of self-assembled CSSP NPs. (**B**) CSSP NPs enhance drug penetration and whole tumor destruction through apoptotic body (ApoBD)-mediated neighboring effect. Reproduced with permission [71]. Copyright 2021, American Association for the Advancement of Science.

Exosomes are a kind of nano-scale membrane structural bodies for intercellular information transmission. Exosomes can selectively extravasate drugs to tumor tissues taking advantage of high permeability and retention effect of solid tumors, improve drug bioavailability and efficacy and reduce side effects. Yang Y et al. [70] isolated exosomes from tumors of advanced cervical cancer patients, loaded CPT onto exosomes by electroporation technology, developed a camptothecin drug delivery platform using tumor-derived exosomes as carriers and showed strong ability to target tumor cells in vitro and patient-derived xenograft (PDX) models. The study demonstrated a substantial enhancement in the sensitivity of tumor cells to ionizing radiation. This was achieved through the regulation of the cell cycle, thereby potentiating the efficacy of radiotherapy. Zhao D et al. [71] formed heterodimer precursor CPT-SS-PR104A via camptothecin (CPT), PR104A and disulfide bond, and then self-assembled into CSSP NPs (Figure 11). Glutathione (GSH) triggered the release of drugs in cells, resulting in the formation of apoptotic bodies containing CPT and PR104A. The apoptosome also delivered drugs to hypoxic tumor cells via proximity effects, achieving deep tumor penetration and thereby eliminating all tumor cell subsets. In vivo experiments on 4T1 tumor models demonstrated the superior antitumor and antimetastatic performance of CSSP NPs, unlike free drugs and simple drug mixtures that failed to achieve robust tumor inhibition due to unsynchronized in vivo drug delivery. Benefiting from effective tumor penetration and hypoxia tumor cell elimination, CSSP NPs also significantly inhibit pulmonary and hepatic metastasis of orthotopic 4T1 tumors. Moreover, CSSP NPs exhibit favorable in vivo biosafety, while free drug treatments cause obvious mouse body weight loss and hepatic damage.

### 3.5. Aptamer-Modified Nanoparticles

**Figure 12:**
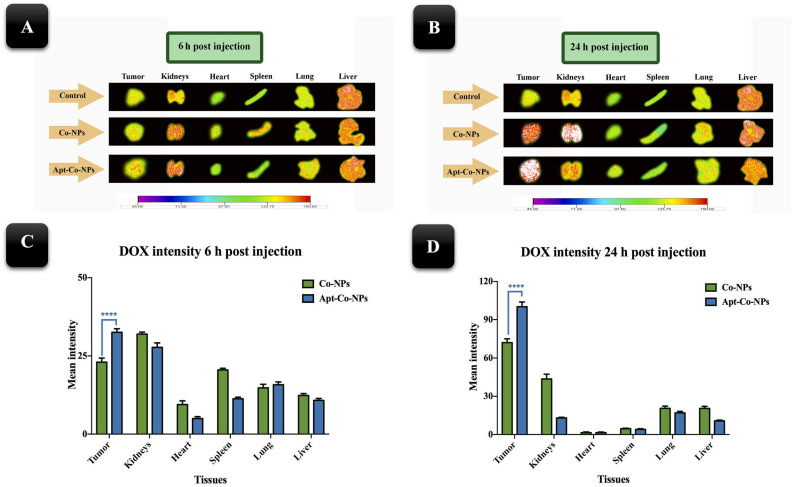
Ex vivo biodistribution of Co-NPs and Apt-Co-NPs (DOX 3 mg/kg + CPT 3 mg/kg) in different tissues (kidneys, heart, spleen, lungs and liver) and tumor of C57BL/6 nude mice 6 and 24 h post-injection, (**A**,**B**) Fluorescence intensity of DOX in these tissues compared with control counterparts (**C**,**D**). All results are displayed as mean ±S.E.M. (*n* = 3). Statistical significance: **** *p* < 0.0001. Reproduced with permission [74]. Copyright 2021, Elsevier B.V.

A plethora of targeting molecules have been developed, including aptamers. Aptamer-modified nanoparticles are a class of functional nanocarriers conjugated with short single-stranded DNA or RNA oligonucleotides screened via the SELEX technology. As high-affinity targeting ligands, aptamers can specifically bind to a wide range of tumor-related biomarkers, which allows nanoparticles to accurately recognize tumor cells and achieve targeted drug delivery. These molecules can be linked to camptothecin via linkers to form aptamer–CPT conjugates (ApDC) or polypeptide-conjugated camptothecin–ApDC for targeted drug delivery [72]. To address the resistance of pancreatic ductal adenocarcinoma (PDAC) to conventional therapies, a sequentially triggered nanoparticle (Apt/CPP-CPTD NPs) was designed for enhanced tumor penetration and intelligent drug release [73]. The nanoparticle features a tenascin-C targeting aptamer (GBI-10) modified on a stroma-permeable cell-penetrating peptide (CPP) for in vivo camouflage and PDAC targeting. In PDAC stroma, tenascin-C detaches GBI-10, exposing CPP to facilitate deeper tumor penetration and cell endocytosis. It has been demonstrated that, upon endocytosis, the high intracellular redox potential triggers controlled drug release. This design enables deep penetration in 3D PDAC spheroids and tumor sections, with mild in vitro cytotoxicity and excellent in vivo antitumor efficacy, demonstrating improved PDAC targeting and reduced systemic toxicity. Shahriari et al. [74] developed a novel nanopolymer constructed from HA and polycaprolactone. This nanopolymer featured an inner aqueous core that encapsulated the hydrophilic drug doxorubicin, while its bilayer structure encapsulated the hydrophobic drug camptothecin. Additionally, the shell surface was functionalized with a FOXM1 DNA aptamer, forming a multifunctional drug delivery system termed Apt-Co-NPs. As shown in Figure 12, co-formulation with or without aptamer produces specific tumor accumulation in vivo, 24 h post-administration. The accumulation of Apt-Co-NPs after 6 and 24 h post injection is higher than that of Co-NPs. In vivo studies in SK-MES-1 nude mice revealed that Apt-Co-NPs achieved a tumor inhibition rate of 87.4%, which was significantly higher than the 74.77% inhibition rate observed with non-targeted Co-NPs. These results suggest that the FOXM1 DNA aptamer enhances the sensitivity of non-small cell lung cancer cells to chemotherapeutic agents, thereby promoting apoptosis and offering a novel strategy for the synergistic treatment of non-small cell lung cancer.

These passive and active targeting abilities of CPT nanoparticles are significant for reducing the side effects and enhancing the efficacy of CPT compounds. Targeted delivery and synergistic therapy systems also show potential to overcome CPT resistance. However, given the complex physiological environment of the body, further exploration and development is required in order to enhance the specific performance of intelligent targeting NPs at tumor sites. It is hypothesized that nanoparticles can achieve precise tumor localization and controllable drug release under the complex physiological microenvironment of tumor tissues, thereby improving antitumor therapeutic efficacy. Such design effectively reduces non-specific accumulation in normal tissues and lowers off-target toxic effects.

## 4. Tumor Microenvironment Responsive Nanomedicine

The tumor microenvironment differs significantly from normal tissue in several aspects: hypoxia, low pH, high GSH levels, high H_2_O_2_ levels, and overexpressed enzymes [75]. These characteristics can all be exploited to develop tumor microenvironment-responsive drugs. This strategy of drug release based on endogenous stimuli and the design of intelligent response drug delivery systems for various tumors is another hot spot in current nanodrug research.

### 4.1. pH-Responsive Nanoparticles

**Figure 13:**
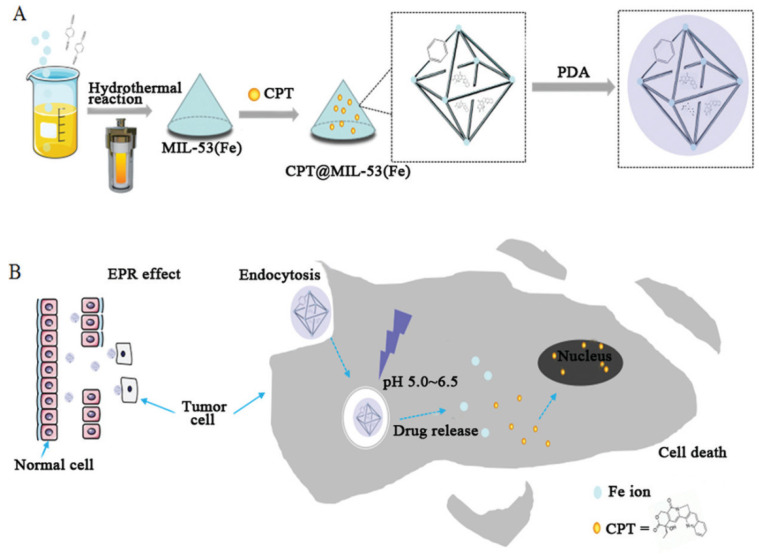
Schematic illustration of the fabrication of PDA@CPT@MIL-53(Fe) (**A**) and its use for MRI-guided pH-sensitive chemotherapy (**B**). Reproduced with permission [79]. Copyright 2022, The Royal Society of Chemistry.

Tumor cells generate large quantities of lactic acid because of their rapid proliferation and augmented glycolytic metabolism, leading to a mildly acidic tumor microenvironment [76,77]. The pH-responsive nanoparticles are nanocarriers designed with pH-sensible groups or chemical bonds that are cleaved in the slightly acidic tumor environment to release drugs [78]. Zhou et al. [79] successfully fabricated a multifunctional theranostic platform of polydopamine (PDA)-functionalized CPT-loaded MOFMIL-53(Fe) nanoparticles with pH-sensitive drug release behavior for MRI imaging guided tumor chemotherapy (Figure 13). The compound’s customary octahedral structure was compromised under acidic conditions, resulting in the effective release of CPT. In an in vivo antitumor assay, the proliferation and metastatic abilities of tumors treated by PDA@CPT@MIL-53(Fe) and free CPT decreased by 93.7% and 94.6% in a zebrafish xenograft model. It demonstrated notable anticancer activity against MCF-7 cells and substantially hindered tumor cell invasion and metastasis in vivo. The introduction of Fe endows PDA@CPT@MIL-53(Fe) with favorable MRI imaging capability, and in vitro tests confirm its excellent MRI performance with a high *r_2_* value of 50 mM^−1^ s^−1^. Collectively, PDA@CPT@MIL-53(Fe) can serve as a promising dual-functional platform for integrated MRI imaging and tumor therapy. Zhang et al. [80] prepared PDA@PCPT nanoparticles by polymerizing pH-sensitive CPT containing polymeric prodrugs (PCPT) and MPC through RAFT strategy and coupling them onto the surface of PDA nanoparticles via amidation. After PDA@PCPT nanoparticles are efficiently internalized by HeLa cells, the cleavage of bifunctional silyl ether bonds in an acidic microenvironment allows for active CPT to be rapidly released, and exhibits remarkable antitumor efficacy in vivo and in vitro after synergistic PTT with clinical transformation potential.

### 4.2. Hypoxia-Responsive Nanoparticles

**Figure 14:**
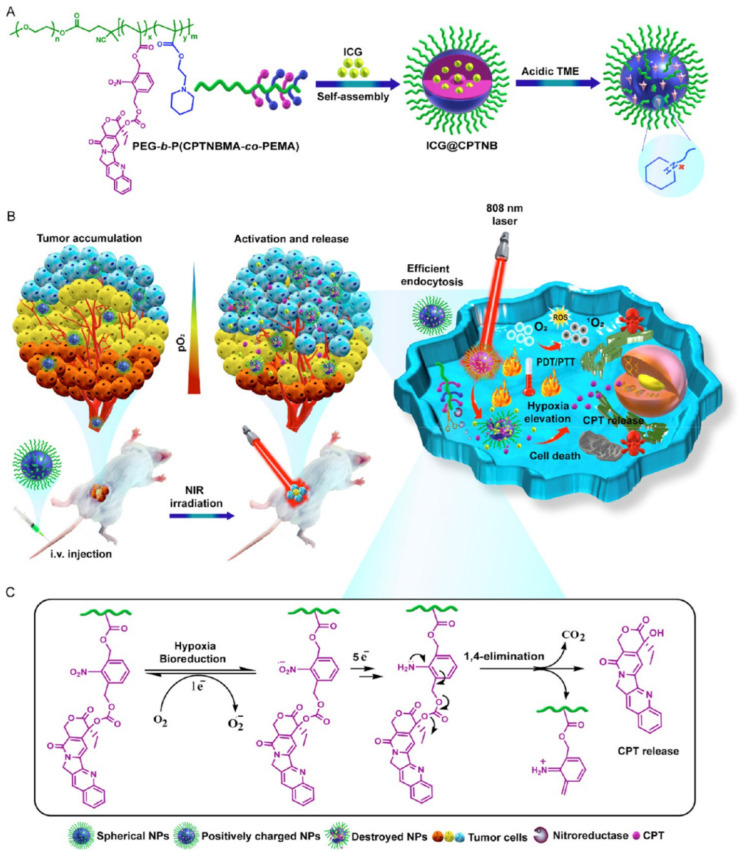
(**A**) Schematic illustration of the fabrication of hypoxia-activatable micellar nanoparticles (NPs) (ICG@CPTNB) and positive-charge transition of ICG@CPTNB in an acidic tumor microenvironment (TME) owing to the protonation of poly(2-methacryloyloxyethyl acrylate) (PMEA) units. (**B**) After intravenous injection, ICG@CPTNB could efficiently accumulate into the tumor. Overall, intratumoral hypoxia was elevated upon NIR irradiation. The positively charged ICG@CPTNB promoted cellular internalization, produced a high amount of singlet oxygen (^1^O_2_), and eventually increased intercellular hypoxia to trigger drug release. (**C**) Mechanistic pathway of CPT release. Reproduced with permission [83]. Copyright 2021, American Chemical Society.

Hypoxia is a general hallmark of malignant tumor growth. Although the tumor site is highly vascularized, these vessels are unable to supply adequate amounts of oxygen and nutrients to all areas, leading to hypoxic tissue in the tumor [81]. Redox nanoparticles targeting local hypoxia could ameliorate local hypoxia by augmenting local oxygen levels [82]. Dutta D et al. [83] reported a hypoxia-activated block copolymer prodrug consisting of PEG and nitrobenzyl linked CPT and PEMA monomer copolymer segments that self-assembled in aqueous solution to encapsulate the indocyanine green (ICG) photosensitizer to yield ICG supported micellar nanoparticles (ICG@CPTNB) (Figure 14). Under NIR laser irradiation, ICG within the nanoparticles generates ROS and local hyperthermia, while the ROS generation process is also highly oxygen consuming, exacerbating hypoxia in the tumor to amplify the release of anoxia-responsive autolytic CPT in the nanoparticles. Photodynamic chemo therapy overcomes the limitations of hypoxia-activated prodrugs or PDT mono therapy and significantly suppresses tumor growth. Moderate tumor inhibition was observed in mice after treatment with free ICG (+L) and free CPT. ICG@CPTNB (−L) performed a significantly higher antitumor efficiency. Most notably, the greatest tumor suppression with the smallest tumor volumes was achieved in the ICG@CPTNB (+L) group. The average ex vivo tumor weights confirmed the synergistic chemotherapy and PDT and PTT amplified antitumor efficacy. These results validated the excellent in vivo antitumor efficacy of ICG@CPTNB by qualifying it to be an excellent candidate for combined photodynamic chemotherapy to realize hypoxic tumor treatment.

Another study, a structurally characterized dual-responsive chemo-photosensitive co-nanoassembly was designed to eradicate primary breast tumors and prevent lung metastasis [84]. This integrated co-nanoassembly is constructed by assembling a biocompatible photosensitive derivative, pheophorbide-diphenylalanine peptide (PPA-DA), with a hypoxia-activated camptothecin (CPT) prodrug, namely (4-nitrophenyl) formate camptothecin (N-CPT). Computational simulations reveal that the co-assembly nanostructure deviates from the conventional core shell configuration and instead comprises numerous small microphase regions. Upon irradiation with a 660 nm laser, PPA-DA generates substantial levels of reactive oxygen species (ROS), effectively inducing apoptosis in normoxic cancer cells. Subsequent to this, the hypoxia-activated N-CPT and CPT infiltrate profoundly into the hypoxic regions of the tumour, thereby curtailing hypoxia-induced tumor metastasis. The antitumor activity of co-nanoassemblies both in the subcutaneous models and orthotopic model were better than the other groups. They accumulated abundantly in tumors, markedly downregulated HIF1α, and potently suppressed lung metastasis via inhibiting metastasis-related proteins. The in vivo antitumor results of both breast cancer models evidently confirmed the safety and synergistic therapeutic outcomes of the co-nanoassemblies. Owing to the rational design of this chemo-photodynamic dual-in-one nanodrug delivery system, this nanomedicine demonstrates significant potential for inhibiting the metastasis of challenging breast tumors.

### 4.3. Redox-Responsive Nanoparticles

**Figure 15:**
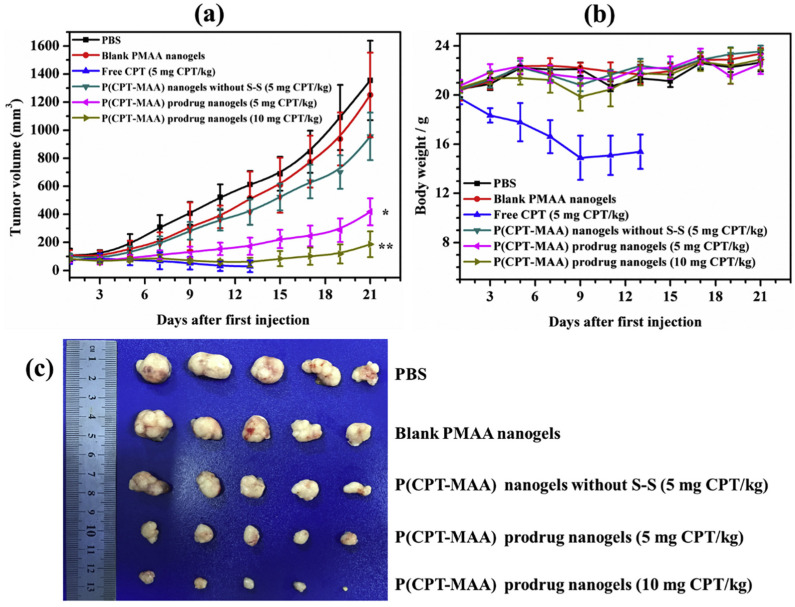
In vivo antitumor assays. Relative tumor volume (**a**) and body weight (**b**) of the Hep G2 tumor-bearing BALB/c nude mice in each group, and photo images of the harvested tumors at 21 days post the first injection (**c**). * *p* < 0.05, ** *p* < 0.01, compared with PBS, Blank PMAA groups. Reproduced with permission [87]. Copyright 2019, Elsevier.

The microenvironment of the solid tumor tissue has a much higher concentration of GSH and ROS in the cytoplasm than in extracellular and blood because of its special origin, nutrition supply, growth morphology and metabolic pathway, leading to a large concentration difference between the extracellular and intracellular microenvironment [85,86]. Ying Qu et al. [87] prepared the P (CPT-MAA) prodrug nanogels by methacrylic acid (MAA), CPT monomer (CPTM) and N,N-methylenebisacrylamide (Bis), in which CPT was covalently incorporated into the nanogels via redox responsive disulfide bonds. The prepared prodrug nanogel (P(CPT-MAA)) exhibited a high concentration of GSH and low pH response characteristics, attributable to the presence of disulfide bonds and PMAA, which possessed redox response characteristics. This property was instrumental in facilitating on-demand drug release in tumor cells and the tumor tissue microenvironment. In addition, P (CPT-MAA) prodrug nanogels exhibited superior antitumor activity in vivo and in vitro. As shown in Figure 15a,c, P(CPT-MAA) prodrug nanogels with 10 mg/kg CPT showed the highest efficiency in the inhibition of tumor growth among all groups. After 21 days injection, the tumor volume (187.3 ± 92.3 mm^3^) was significantly lower than that of the mice treated by PBS (1354.4 ± 283.3 mm^3^, *p* < 0.01). Free CPT caused severe adverse reactions: obvious pain, a 22% body weight loss and 100% mortality within 13 days at 5 mg/kg (Figure 15b). By contrast, no significant toxic side effects were observed in P(CPT-MAA) prodrug nanogels, showing greatly improved biosafety. It might be a promising drug delivery system.

Xu Hongting et al. [88] prepared HA-SS-MTX-LA@HCPT NPs loaded with HCPT by HA-disulfide-methotrexate-linoleic acid polymer nanoparticles. In vitro release test indicated that the NPs could rapidly release drugs under the condition of high concentration GSH, MTX and HCPT exerted synergistic killing effect on HepG2 cells and Bel-7402 cells through different mechanisms and improved curative effect. Zhang et al. [89] first connected the 10-phenolic hydroxyl group of 7-ethyl-10-hydroxycamptothecin (SN38) with short-chain OEG through thioether bond, and successfully obtained the nano-delivery system OEG-2S-SN38 with GSH/ROS dual-responsive spherical vesicle structure, which could rapidly release SN38 through thiolysis in cells with high GSH expression and oxidative hydrolysis in cells with high ROS expression. OEG-2S-SN38 showed better antitumor activity than CPT-11 in various tumor cell lines cultured in vitro, BCap37 human breast cancer xenografts in nude mice and orthotopic colon cancer models, which deserved further study.

### 4.4. Enzyme-Responsive Nanoparticles

**Figure 16:**
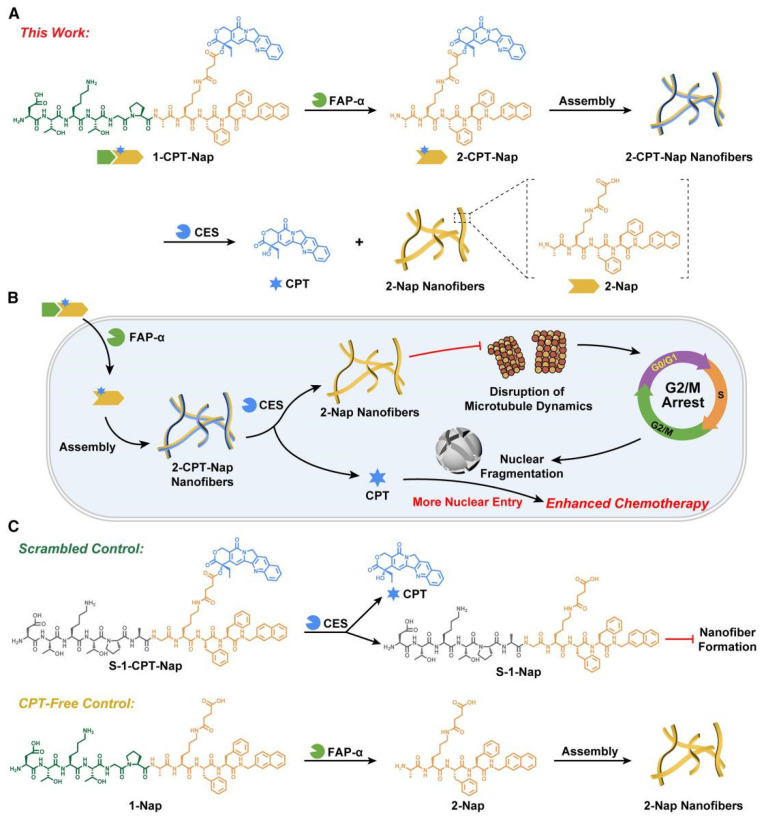
Chemical evolution and working mechanism of 1-CPT-Nap, the scrambled control S-1-CPT-Nap, and the CPT-free control 1-Nap. (**A**) Molecular structure of 1-CPT-Nap and its chemical evolution. (**B**) Schematic illustration of 1-CPT-Nap for enhancing the nuclear delivery of CPT. (**C**) Molecular structures of S-1-CPT-Nap and 1-Nap and their chemical evolutions. Reproduced with permission [93]. Copyright 2025, Elsevier Inc.

Under pathological conditions such as tumor or inflammation, the over-expression of certain enzymes plays an important role in tumor cell proliferation, angiogenesis, invasion and metastasis [90]. Researchers took advantage of the high specificity of enzymes to develop enzyme-responsive nanodrug delivery systems. Enzyme-responsive smart nanoparticles selectively interact with enzymes overexpressed in tumor tissues under mild conditions, leading to structural changes such as disassembly, morphological transformation, charge reversal and covalent bond cleavage [91]. Such behaviors facilitate targeted drug delivery in tumors and lower systemic toxicity. Among them, matrix metalloproteinases (MMPs) are one of the most extensively studied proteases that can catalyze the degradation of nanoparticles and facilitate drug penetration in tumor tissues. Yang et al. [92] established a connection between a MMP-2 cleavable peptide sequence (GPLGVRGE) and a hydrophobic near-infrared dye (Cy5) to create an amphiphilic multifunctional molecule (Pep-Cy5). This molecule was capable of self-assembling with hydrophobic antitumor drugs (camptothecin and trans-retinoic acid) to form water-soluble enzyme-responsive nanoparticles. These NPs showed an ideal MMP-2-triggered degradation process. The MMP-2-induced degradation and hydrophobic antitumor drug interchangeability features of this nanosystem enable the hydrophobic antitumor drugs to exhibit longer blood-retention times, improved intratumoral accumulation, fewer side effects, and higher anticancer efficacies compared with free drugs. Self-assembled nanodrugs produced prominent antitumor effects, with an inhibition rate of 69.9%. By comparison, the rates for RA, CP and CP/RA combination were 41.2%, 53.1% and 59.3%, respectively. This new MMP-2-responsive self-assemble strategy offers a new approach to improving the biological compatibilities and antitumor efficacies of chemotherapy drugs while reducing adverse effects. A recent study [93] introduced a novel strategy to enhance the efficacy of CPT against pancreatic cancer by leveraging intracellular nanofiber formation and nuclear fragmentation (Figure 16). The researchers designed a drug–peptide conjugate, Asp-Thr-Lys-Thr-Gly-Pro-Ala-Lys(SA-CPT)-Phe-Phe-Nap (denoted as 1-CPT-Nap) with four key components: a hydrophilic tetrapeptide for solubility enhancement, a sequence for fibroblast activation protein alpha (FAP-α) cleavage, a self-assembling motif, and an anticancer alkaloid CPT. It upon activation by FAP-α and carboxylesterase, releases CPT and simultaneously assembles into Ala-Lys(SA)-Phe-Phe-Nap (2-Nap) nanofibers inside cells. These in situ-formed 2-Nap nanofibers induce cell cycle arrest at the G2/M phase and subsequent nuclear fragmentation, thereby facilitating enhanced nuclear entry of CPT. Cellular experiments demonstrate that the nuclear fragmentation caused by 2-Nap nanofibers results in a 1.8-fold increase in CPT accumulation within nuclei. In vivo studies further reveal that the antitumor efficacy of 1-CPT-Nap against MIA PaCa-2 pancreatic tumor models is 1.5 times greater than that of free CPT. This strategy of intracellular enzymatic self-assembly into nanofibers offers a promising approach for improving nuclear delivery of chemotherapeutic agents.

Despite considerable progress in the design and implementation of tumor microenvironment-responsive nanoparticles, the development of a universal antitumor nano-delivery platform remains challenging due to the complexity and heterogeneity of the tumor microenvironment. The in vivo action mechanism of endogenous responsive nanoparticles remains incompletely elucidated. Further in-depth investigations are still required to promote their clinical translation and practical application. Moreover, a single endogenous stimulus response is insufficient to satisfy the requirement of precise tumor therapy. As a result, the development of comprehensive intelligent drug delivery system combining with exogenous stimuli becomes a crucial research direction in the future, which requires urgent wisdom and efforts from researchers.

## 5. Exogenous Stimulus-Responsive Nanodrugs

Some biomaterials exhibit specific responsiveness to exogenous stimuli, such as light, ultrasound, magnetic fields, and X-rays. The integration of these functional materials with camptothecin compounds can construct exogenous stimulus-responsive nanoplatforms that achieve precise on-demand drug release in tumor tissues. Such spatiotemporally controllable drug release effectively enhances the antitumor efficacy and mitigates the systemic side effects of camptothecin [94,95,96,97,98]. This on-demand release strategy offers great potential for the development of novel nanopharmaceuticals. Collectively, this rational design approach represents one of the most promising strategies for precise tumor therapy.

### 5.1. Photo-Thermal-Responsive Nanoparticles

PTT using photothermal conversion agents and NIR laser to ablate tumors has drawn extensive concern as a novel strategy for tumor treatment. Its principle of action is to utilize photothermal converters to absorb NIR and convert it into high heat to kill tumor cells [99]. PTT has been demonstrated to exhibit a wide range of applications, non-invasive properties, strong selectivity, and minimal damage to normal tissues. These characteristics suggest significant potential for use in tumor therapy and drug release control [100]. Yang et al. [101] developed a simple and smart pH/NIR dual-stimulus-responsive degradable mesoporous CoFe2O4@PDA@ZIF-8 sandwich nanocomposite. The mesoporous CoFe2O4core acts as T2-weighted magnetic resonance (MR) imaging probe, PTT agent, and loading platform of hydrophilic doxorubicin (DOX). A PDA layer is used to avoid the premature leakage of DOX before arriving at tumor site, enhance PTT efficiency, and facilitate the integration of ZIF-8 (a kind of metal-organic framework). The ZIF-8 shell serves to encapsulate hydrophobic camptothecin and as the switch for the pH and NIR stimulation-responsive release of the two drugs. Therefore, the burst release of both drugs under acidic condition and NIR stimulation, T2-Mr imaging guided chemotherapy and PTT cotherapy enhances the antitumor efficacy of monotherapy and reduces chemotherapy side effects. The combination of chemotherapy and PTT is a very ideal approach for tumor treatment and has good application prospects in tumor treatment. Another study [102] reported the development of an amphiphilic drug–drug conjugate (IR820-SS-CPT) by linking CPT with the photothermal agent IR820 via a redox-responsive disulfide linker. This conjugate self-assembles into nanoparticles (IR820-SS-CPT NPs) in aqueous solution, enhancing the membrane permeability of IR820 and the aqueous solubility of CPT. The disulfide bond enables on-demand drug release in the GSH-rich tumor microenvironment. Notably, IR820-SS-CPT NPs achieved nearly 100% therapeutic agent loading efficiency and demonstrated significant tumor cell killing efficiency in vitro and antitumor effects in vivo. This design offers a promising nanotherapeutic platform for chemo-PTT.

### 5.2. Ultrasonically Triggered Nanoparticles

**Figure 17:**
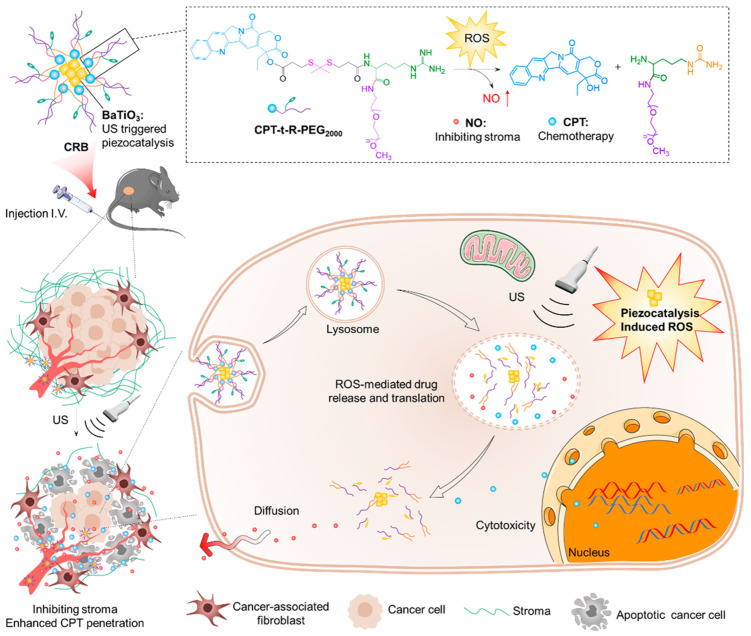
Schematic illustration of the effective tumor chemotherapy with inhibiting stroma and enhancing CPT penetration through US triggered piezocatalysis and nanoprodrug strategy. Reproduced with permission [107]. Copyright 2023, American Chemical Society.

Ultrasound-triggered nanoparticles harness mechanical energy from high-frequency ultrasound (sound waves, radiation pressure, and heat) to induce vibration, cavitation, or mechanochemical disruption of drug-loaded microbubbles, enabling molecular-level rearrangement or bond cleavage at targeted sites for spatiotemporally controlled drug release [103] and precise modulation of tissue penetration depth and energy [104]. Zhu et al. [105] developed novel 10-HCPT loaded tumor homing peptide functionalized drug-loaded phase change nanoparticles (tLyP-1-10-HCPT-PFP NPs) for low intensity focused ultrasound (LIFU) assisted molecular imaging and precise tumor treatment. tLyP-1 peptide can penetrate tumor blood vessels and stroma to guide nanoparticles deep into tumor tissue and into cytoplasm. With the help of LIFU, nanoparticles can enhance tumor ultrasound molecular imaging by phase conversion, cause intracellular explosion and release of 10-HCPT to significantly inhibit tumor growth. Huo et al. [106] synthesized PCPT by controlled radical polymerization with POEGMA as the main disulfide group and PCPT as anticancer drug through coupling of beta-site disulfide group and carbonic acid bond. Ultrasound-induced radiation can cleave disulfide groups to stimulate intramolecular cyclization and release CPT activating drugs, which has obvious inhibitory effect on Hela cells. Wang et al. [107] involved the conception of an ultrasound-responsive nano-prodrug, designated CPT-tR-PEG2000@BaTiO_3_ (CRB). This nano-prodrug consists of an amphiphilic prodrug molecule, comprising piezoelectric nanomaterial barium titanate (BaTiO_3_) and camptothecin (CPT), linked by thioketal (TK) bonds (t) and NO donor L-arginine (R) (Figure 17). Under ultrasound trigger, BaTiO_3_ in CRB can continuously generate ROS in tumor hypoxia environment to trigger cascade reaction to break sulfur ketal bond to release CPT and oxidize R to release NO. This process not only delivers CPT and NO to the tumor site, but also depletes dense stroma, enhances CPT delivery and therapeutic effect, reduces chemotherapy resistance, and significantly improves the antitumor efficacy of highly malignant Panc02 tumors in mice. The PBS group showed an 8.47-fold increase in tumor volume, with no tumor inhibition. In contrast, CRB nanoparticles plus US treatment only led to a 3.8-fold tumor volume increase, exerting the most potent antitumor efficacy across all groups. Overall, this chemical design of CRB nanoprodrug provided a practicable approach for targeted therapy against Panc02 and other PDACs.

### 5.3. Magnetic Nanoparticles

Magnetic nanoparticles have garnered considerable attention in biological and biomedical fields, with wide applications including MRI contrast agents, magnetic hyperthermia and targeted drug delivery. Targeted anticancer drugs delivery and release by magnetic nanocomposites is a special and potential application [108]. It is anticipated to be a safe and less side-effect medical technique to satisfy the requirements of clinical application. Seong Deok Konga et al. [109] reported a lipid polymer hybrid nanoparticle system SRNPs loaded with camptothecin (CPT) and ferroferric oxide (Fe_3_O_4_). Lipid polymer nanoparticles can control the release of CPT by utilizing a remote radio frequency (RF) magnetic field. The nanoparticles significantly inhibited the growth of MT2 mouse breast cancer cells in vitro in the presence of remote RF field. Ayat A Allam [110] incorporated CPT into a lipid mixture of 1,2-dipalmitoyl-sn-glycerol-3-phosphocholine (DPPC) and l-α-dipalmitoylphosphatidyl glycerol (DPPG) via biotin-avidin interaction and subsequently immobilized them onto the surface of superparamagnetic nanoparticles Fe_3_O_4_ to prepare magnetocaloric responsive nanocomposite Superparamagnetic Iron Oxide Nanoparticle (SPION) with high loading rate of CPT which enhanced the solubility and stability of CPT. Magnetically induced hyperthermia can be exploited to achieve stimulation-induced CPT release from lipid-encapsulated NPs. Magnetic hyperthermia and chemotherapy induced by SPION have synergistic effect and are more cytotoxic to acute T cell leukemia jurkat cells than CPT monomer.

### 5.4. X-Ray-Responsive Nanoparticles and Micro-Electric Fields

**Figure 18:**
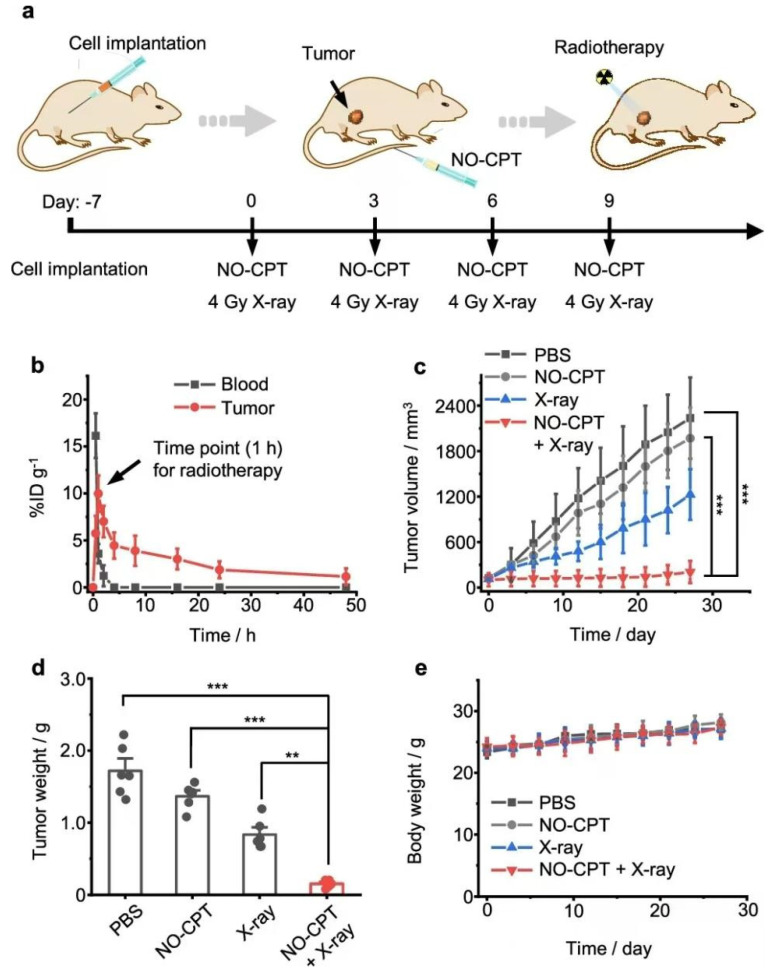
Radiotherapy activates a prodrug that rejects tumor growth. (**a**) Treatment scheme. Nu/Nu mice were implanted subcutaneously with HCT116 cells, followed by intravenous (iv) injection of NOCPT (10 mg/kg) and radiotherapy (4 Gy each treatment). (**b**) Time-dependent accumulation of NO-CPT in tumor tissue detected by UPLC-UV (*n* = 3). (**c**) Tumor growth curves of tumor-bearing mice after the indicated treatments. (**d**) Average tumor weight at day 27 after the first treatment. (**e**) Weight change curves of mice subjected to different treatments. *n* = 6, two-tailed unpaired Student’s *t*-test, ** *p* < 0.01, *** *p* < 0.001. Reproduced with permission [111]. Copyright 2022, American Chemical Society (ACS).

Other CPT nanomedicines such as those responsive to electric field and X-ray stimuli have also been reported. Recent advancements in prodrug activation strategies have been reported, highlighting the innovative use of radiotherapy (X-ray) to activate prodrugs [111]. The precision and deep tissue penetration of X-ray make it an ideal tool for altering molecules within tumors through water radiolysis. Studies have demonstrated that N-oxides can be effectively reduced by hydrated electrons (e-aq) generated from radiation, both in vitro and in living cells. This mechanism has been successfully applied to activate N-oxide prodrugs, with a notable example being the camptothecin (CPT)-based N-oxide prodrug, which exhibits a remarkable anticancer effect upon activation by radiotherapy (Figure 18). This radiation-induced in vivo chemistry opens up versatile possibilities for designing radiotherapy-activated prodrugs, potentially revolutionizing cancer treatment strategies. A novel approach has been presented involving tumor pH and ROS-responsive polyprodrug micelles loaded with the X-ray photosensitizer verteporfin (VP) to enhance ROS production [112]. The block copolymer polyprodrug, composed of hydrophilic poly(ethylene glycol) (PEG) and segments of thioketal-linked camptothecin (CPT) methacrylate (CPTKMA) and 2-(pentamethyleneimino)ethyl methacrylate (PEMA) (PEG-b-P(CPTKMA-co-PEMA)), can self-assemble into micelles in aqueous solution and encapsulate VP with a high loading efficiency of 67%. Within the tumor microenvironment, the micelles’ zeta potential shifts from neutral to positive under acidic conditions, promoting cellular internalization. Upon X-ray irradiation at 4 Gy, the presence of VP significantly enhances ROS generation, which in turn triggers the release of CPT. This synergistic radiochemotherapy, driven by deep X-ray penetration, ROS generation enhancement, and triggered CPT release, effectively ablates tumors. This innovative strategy highlights the potential of X-ray-triggered activation of CPT prodrugs as a robust therapeutic modality for enhanced cancer radiochemotherapy.

### 5.5. Pyroelectric Potential-Mediated Therapy

Self-propelled and field-responsive nanoplatforms address poor tumor penetration and intracellular drug delivery. Thermally induced pyroelectric and thermophoretic effects endow nanoparticles with active mobility and electrical activation, boosting synergistic antitumor efficacy. A study proposes in situ generation of micro-electric fields on nanoparticle (NP) surfaces, removing the need for external electric fields to overcome drug penetration barriers in tumors [113]. Researchers synthesized Janus tBT@PDA-CPT NPs, using pyroelectric tetragonal barium titanate (tBT) NPs as the core substrate. Camptothecin (CPT) was conjugated to the NPs via disulfide bonds. Under NIR irradiation, the PDA hemisphere of the Janus NPs generates an asymmetric thermophoretic force. This force propels NP motion, enhancing tumor accumulation, deep tissue penetration, and cellular interactions. Photothermal heating of the tBT NPs induces temperature fluctuations, triggering a pyroelectric potential. This potential selectively alters the membrane potential of tumor cells while sparing normal cells, significantly enhancing tumor cell internalization and cytotoxicity. Comprehensive analysis confirmed synergy among pyroelectric dynamic therapy (PEDT), chemotherapy, and PTT. This combined approach drastically reduced the required CPT dosage, achieving potent tumor growth suppression and significantly prolonging animal survival in vivo. Leveraging inherent differences in membrane potential and GSH levels between tumor and normal cells, this study demonstrates a streamlined design. It enables thermophoretic-driven NP motility, pyroelectric potential-enhanced cellular internalization, and synergistic PTT/PEDT/chemotherapy for antitumor treatment.

However, there are still some issues such as low selectivity, low drug loading capacity, drug leakage and toxic side effects in the drug release control by external activation. Thus, it remains a valuable research hotspot to investigate new techniques to effectively address these issues and realize remote control of drug active regions.

## 6. Clinical Application Status

Despite the extensive studies on CPT nanodrugs, few have found their way into clinical use. Current CPT nanopharmaceuticals in clinical use are primarily based on semisynthetic CPT derivatives such as irinotecan and SN38. Liposomal irinotecan (Onivyde) has been approved in numerous countries and is used in combination with 5-fluorouracil and leucovorin for the treatment of metastatic pancreatic cancer [114]. SN-38 Polymeric Micelle (NK012), produced by covalent coupling of SN38 and PEG-b-P(Glu) polymer developed by Kyowa Fermentation Kirin Co Ltd. in Japan through ester linkage, remains in clinical study for the treatment of various cancers including colorectal cancer, small cell lung cancer and triple negative breast cancer [115,116]. DEP irinotecan is a dendrimer prodrug developed by StarPharma in Australia that covalently couples SN-38 to a PEGylated polylysine dendrimer via a hydrolytically labile linker [117] and has been used in clinical studies of colorectal cancer. Additionally, antibody-drug conjugates (ADC) made from semisynthetic CPT derivatives have also shown promise. Sacituzumab Govitecan (Trodelvy, IMMU-132) developed by Immunomedics Inc. in the USA is obtained by coupling monoclonal antibody hRS7 targeting Trop-2 on cancer cell surface with SN-38 through acid-sensitive degradable linker CL2A. In phase II clinical trials, pretreated mTNBC patients treated with this drug had an overall response rate of 33.3% and a clinical benefit rate of 45.4%, demonstrating significant efficacy [118]. It was approved for triple negative breast cancer treatment in 2020 [119]. Deruxtecan (DS-8201) was developed in collaboration with AstraZeneca in the UK by the First Third Republic of Japan and is a conjugate of DXd (a derivative of Exatecan) to trastuzumab via an enzyme-cleavable tetrapeptide (GGFG) linker and cysteine and aminomethyl groups. It has been approved for the treatment of breast cancer, metastatic gastric cancer and non-small cell lung cancer [120]. DS-8201 has sparked a surge in the development and transformation of camptothecin payloads by many domestic companies. With the release of clinical data on site-specific conjugation technology and other payload-like products, ADC is expected to deliver more innovations and breakthroughs. LY01616, a new antitumor drug developed by Nanjing Luye Pharmaceutical, is a combination preparation of irinotecan and fluorouracil in the same liposome. As demonstrated in preclinical trials, this formulation has been shown to maintain drug synergy, enhance therapeutic efficacy and reduce toxic side effects [121]. It is currently undergoing phase I/II clinical trials. CRLX101 is a nanoparticle composed of cyclodextrin and 20(S)-camptothecin, which is currently undergoing phase IIa clinical trials as a potent inhibitor of topoisomerase 1 (topo-1) and hypoxia-inducible factor-1 (HIF-1). Clinical data shows that it has superior pharmacokinetic properties, good tolerability, enhanced pharmacodynamics and superior efficacy to camptothecin [122]. VIP236 is an intelligent carrier drug containing a peptide mimetic module targetingαvβ3integrin (containing RGD159) conjugated to 7-Ethyl Camptothecin payload via a linker containing neutrophil (NE) cleavage site. It can specifically target αvβ3integrin of tumor cells and then be effectively cleaved by neutrophil elastase (NE), achieving excellent antitumor effect in xenograft (PDX) cancer models such as triple negative breast cancer, renal cell carcinoma and colorectal cancer [123]. The first human study in patients with advanced or metastatic solid tumors is currently underway. BL0020 is obtained by coupling 7-ethyl-10-hydroxycamptothecin (SN-38), linker and PEG modified *ε-poly-L-lysine*. Preclinical studies have shown that BL0020 has great potential in the treatment of refractory malignancies such as pancreatic cancer, small cell lung cancer, liver cancer, breast cancer and soft tissue sarcoma. BL0020 is currently in phase I clinical trials in China and Australia [124]. LE-SN38 liposomes were developed in 2005 and pre-clinical studies have demonstrated good safety and efficacy. However, in phase II clinical trials for metastatic colorectal cancer, the anticipated therapeutic effect was not achieved [125]. In conclusion, notwithstanding the challenges inherent in the development of CPT nanodrugs, their increasingly evident potential for clinical application is worthy of note. Recent advancements in the clinical investigation of CPT nanodrug delivery systems have led to the anticipation that CPT nanodrugs will assume an increasingly prominent role in future cancer treatment modalities.

## 7. Multidimensional Assessment and Clinical Adaptability of Camptothecin-Based Nanoformulations

### 7.1. Comprehensive Comparison and Advantage–Disadvantage Analysis

When systematically evaluating the clinical translation potential of camptothecin and its derivative nano-delivery systems, individual formulation research and performance characterization can no longer meet the requirements of pharmaceutical research, development and clinical decision-making. Therefore, it is essential to systematically evaluate various nano-delivery platforms from five core dimensions, including drug loading capacity, targeting efficiency, controllable release performance, preparation feasibility and biological safety. This comprehensive analysis helps clarify the inherent strengths and limitations of different nanosystems, as well as their clinical translation bottlenecks and suitable application scenarios. This provides evidence-based support for the design of nanoformulations for camptothecin compounds.

From the perspective of drug loading capacity, inorganic nano-carriers demonstrate significant advantages due to their structural characteristics. Taking porous materials such as metal–organic frameworks (MOFs) and mesoporous silica nanoparticles (MSNs) as representatives, their precisely tunable ordered mesoporous channels (pore size 2–50 nm), extremely high specific surface area (>1000 m^2^/g), and abundant surface modification sites enable drug loading capacities reaching 30–50 wt%, significantly higher than liposomes at 5–10 wt% and polymeric micelles at 10–20 wt% [126,127]. This can satisfy drug delivery demands in high-intensity treatment scenarios such as high-dose chemotherapy and combination therapy. However, inorganic carriers such as MSNs and MOFs generally exhibit poor biodegradability, which is a difficult-to-overcome clinical translation constraint. They are easily captured by the reticuloendothelial system (RES) in vivo and accumulate long-term in organs such as the liver, spleen, and lungs, continuously stimulating immune cell activation, inducing chronic inflammation, oxidative stress, and potential immunotoxicity. Their long-term safety has not yet been clinically validated, greatly limiting applications in systemic administration scenarios. In contrast, although liposomes have relatively low drug loading efficiency, their phospholipid bilayer structure is highly homologous to biological cell membranes, possessing excellent biocompatibility and low immunogenicity. Meanwhile, liposome preparation technology is mature, with strong controllability of key parameters such as particle size, encapsulation efficiency, and stability, and has been fully adapted to GMP industrial production systems. They are currently the most successful and evidence-supported nano-delivery platform for the clinical translation of camptothecin drugs. The development and approval history of irinotecan liposome (Onivyde^®^) serves as a typical example. Polymeric micelles occupy a medium level in drug loading capacity (10–20 wt%). Their hydrophobic core can accommodate camptothecin molecules, and the preparation process requires no organic solvents, with low cost and relatively simple process. However, the limited drug loading capacity may become a constraining factor in high-dose demand scenarios for camptothecin. In addition, some polymeric materials may trigger complement activation and inflammatory reactions, requiring careful assessment of the superimposed effect between the carrier itself and camptothecin toxicity. Nucleic acid drugs have limited application as carriers in camptothecin delivery. Their main value lies in gene therapy combination strategies, such as co-loading siRNA to reverse drug resistance gene expression, rather than directly serving as camptothecin loading platforms. Their storage conditions are harsh, in vivo stability is poor, and the synergistic mechanism with camptothecin still requires in-depth validation. Exosomes have extremely low drug loading efficiency (usually <5 wt%), and encapsulation of highly hydrophobic camptothecin molecules is particularly difficult. Although their natural targeting ability and low immunogenicity have theoretical appeal, preparation processes are complex. And they currently remain at the proof-of-concept stage, with substantial barriers to clinical translation.

From the perspective of industrial production and scale-up transformation, liposomes and PLGA-based polymeric nanoparticles have achieved mature GMP-level industrial production, with standardized preparation processes and well-established quality control systems. Moreover, the FDA has approved multiple PLGA nanoformulations for marketing, with fully validated process safety and compliance, possessing core advantages for rapid clinical translation. In contrast, nucleic acid nano-structures, MOF/COF-based inorganic-organic hybrid carriers, and multi-functional intelligent delivery systems rely on multi-step organic reactions, stringent conditions, and complex surface functionalization modifications for their synthesis. They impose extremely high technical requirements on preparation equipment, environment, and personnel, constituting fundamental obstacles to their clinical translation.

Targeting efficiency is a core indicator determining the tumor enrichment capability of nanoformulations. The preclinical–clinical translation difference of targeting strategies is a key controversy in the current field. Theoretically, ligand-modified active targeting nanoparticles such as folate, transferrin, HA, and peptides can specifically recognize highly expressed targets on tumor cell surfaces through receptor-mediated endocytosis, with tumor cell uptake efficiency 3–5 times higher than passive targeting systems relying solely on the EPR effect. They demonstrate stronger tumor enrichment and antitumor effects in preclinical models such as subcutaneous xenografts and orthotopic tumors. However, upon entering the complex in vivo physiological environment, ligand modification increases nanoparticle surface molecular weight, steric hindrance, and charge heterogeneity, accelerating non-specific clearance by the mononuclear-phagocyte system, shortening drug in vivo circulation half-life, and partially offsetting the targeting enrichment advantage [128]. Meanwhile, tumor heterogeneity leads to significant differences in target expression among different patients and different tumor stages, with obvious individual differences in ligand–receptor binding efficiency. This demonstrates that preclinical targeting advantages cannot be directly translated into clinical survival benefits. Target specificity, in vivo stability, immune clearance, and multiple other factors constitute the core translational barriers of active targeting systems. Nevertheless, the approval of two camptothecin derivative-based ADCs—sacituzumab govitecan (Trodelvy^®^, SN-38 payload, anti-TROP-2) and trastuzumab deruxtecan (Enhertu^®^, DXd payload, anti-HER2)—offers promising prospects for the clinical translation of camptothecin active-targeting nanoformulations.

At the level of release controllability, stimulus-responsive delivery systems based on tumor microenvironment characteristics (pH, GSH, enzyme, ROS triggers) represent the frontier direction of precision drug release. However, such intelligent systems still have significant shortcomings: multi-step chemical modifications and complex responsive group coupling lead to cumbersome preparation processes, with poor batch-to-batch reproducibility in particle size, encapsulation efficiency, and response thresholds. Moreover, different tumor microenvironments exhibit high heterogeneity in pH, redox levels, and enzyme activity, easily causing uncontrollable release behavior, constraining their clinical implementation.

Thus, it is evident that no universally optimal nano-delivery platform exists. Formulation screening and design must be anchored to clinical demand priorities, achieving precise performance-scenario matching.

### 7.2. Precision Matching of Camptothecin Nanoformulations to Clinical Demands

Based on the above multi-dimensional quantitative comparison and bottleneck analysis, more targeted rational design strategies for camptothecin nanoformulations can be proposed for different clinical application scenarios, achieving high coupling between technical performance and clinical value. For solid tumors with poor prognosis urgently requiring rapid clinical translation, such as pancreatic cancer and small cell lung cancer, liposomes and albumin nanoparticles are the optimal choices. Onivyde^®^ has confirmed the survival benefits of liposomal irinotecan in pancreatic cancer treatment. Meanwhile, albumin nanoparticles rely on the mature delivery platform of Abraxane^®^ and its long-circulating properties in vivo to enable rapid formulation transformation of camptothecin derivatives.

For clinical scenarios requiring high drug loading, such as dose-intensive chemotherapy and multi-drug combination therapy, mesoporous silica, MOF/COF porous materials, or carrier-free nanocrystals possess unique advantages. Especially, carrier-free nanocrystals are formed through self-assembly of drug molecules, with theoretical drug loading capacity approaching 100%, completely avoiding carrier-related toxicity while simplifying the preparation process.

In the treatment of central nervous system tumors such as high-grade gliomas requiring blood-brain barrier (BBB) penetration, traditional passive targeting completely fails. The BBB structure in infiltrating regions of high-grade gliomas remains intact, and the EPR effect is negligible. Active targeting must be relied upon to achieve transcellular transport. Transferrin receptor (TfR) and lactoferrin receptor (LfR) are highly expressed on both brain microvascular endothelial cells and glioma cell surfaces, providing a molecular basis for dual-targeted delivery. Lactoferrin-modified camptothecin nanoparticles can efficiently penetrate the BBB through receptor-mediated transcytosis-exocytosis pathways, with brain drug accumulation 3–5 times higher than passive targeting systems, significantly enhancing the antitumor effect in orthotopic gliomas [129]. For P-glycoprotein (P-gp)-mediated multidrug resistance (MDR) tumors, single camptothecin delivery is insufficient to reverse drug efflux, and multi-drug co-delivery strategies become the core solution. Co-loading P-gp inhibitors and camptothecin in nanoparticles can inhibit drug efflux, significantly increase intracellular drug concentration, and reverse the drug-resistant phenotype.

In frontier theranostics scenarios, inorganic nano-carriers such as gold nanoparticles, mesoporous manganese dioxide, and upconversion nanoparticles stand out due to their unique optical, magnetic, and photothermal properties: gold nanoparticles can achieve CT imaging-guided photothermal combination therapy, mesoporous manganese dioxide can achieve magnetic resonance imaging (MRI) and chemodynamic therapy, and upconversion nanoparticles rely on NIR deep tissue penetration capability to achieve precise tumor imaging and photodynamic-chemotherapy synergistic therapy, simultaneously completing lesion visualization monitoring and therapeutic intervention. However, the long-term biosafety of such inorganic carriers remains a major shortcoming in the field. Their in vivo metabolic pathways are unclear; they easily accumulate long-term in the liver and spleen, and long-term toxicity risks are unknown, constraining clinical application.

For scenarios such as oral administration in colorectal cancer and postoperative maintenance therapy, polymeric nanoparticles and cationic cyclodextrin inclusion complexes possess natural advantages. Cationic cyclodextrin can stably encapsulate camptothecin through electrostatic interactions, resist gastrointestinal acid–base environments and digestive enzyme degradation, and achieve specific accumulation in the colon. For complex clinical demands requiring spatiotemporal precision release control and maximized reduction of systemic toxicity, multi-responsive intelligent delivery systems (pH/ROS/enzyme) represent the core future development direction, capable of achieving stable circulation in normal tissues and rapid drug release in the tumor microenvironment.

In summary, the formulation selection and direction for camptothecin and its derivative nano-delivery systems should strictly follow the core principle of prioritizing clinical needs over technological novelty, abandoning approaches that purely pursue structural complexity and flashy functions.

## 8. Clinical Transformation Challenges and Future Prospects

### 8.1. Problems and Challenges in Current Research

CPT compound-loaded NPs are classified differently and have different characteristics depending on the material (Table 2). CPT compound-based nanoformulations have demonstrated significant therapeutic effects in the biomedical field, but the current clinical conversion rate is slow and faces many challenges, including formulation factors, biological factors and regulatory considerations.

#### 8.1.1. Nanoformulation Factors

Passive targeting nanoparticles are easy to synthesize and take advantage of enhanced permeation and retention (EPR) effect but without specificity; active targeting provides better accuracy but it encounters obstacles such as complex synthesis and heterogeneous expression of receptors. Nanoconjugates represented by ADC have made some advances, however, it is not easy to develop antibodies to new targets, most of them focus on conventional targets such as Her-2 and Trop-2, and the delivery efficiency and tumor penetration depth still need improvement. The clinical translation of stimulus-responsive prodrug strategies still suffers from the issues of complicated prodrug design, low targeting efficiency in vivo and uneven activation and dispersion. In terms of formulation manufacturing, the large-scale manufacturing of nanoformulations needs to ensure high quality and batch-to-batch reproducibility. While basic nanosystems can be manufactured successfully at scale, complex nanosystems (surface modifications, inclusion of targeted elements, or encapsulation of multiple drugs) are more difficult to manufacture, increasing the complexity and cost of large-scale manufacturing and complicating quality assurance and assessment procedures. Particle size is a crucial factor influencing nanoparticle behavior in vivo, and nanoparticles should be developed at least above 10 nm to avoid renal clearance and below the uptake cut-off values for vascular fenestrations (100–175 nm) and liver and spleen endothelial slits (200–500 nm) [130,131,132]. Nanoparticles are commonly developed in the 100–200 nm range to deliver drugs to solid tumors via the phenomenon of EPR, but tumor vascular heterogeneity can result in differences in drug distribution and therapeutic efficacy. Furthermore, when the load of the pharmaceutical compound is low, a large quantity of nanoparticles is administered in order to achieve the desired therapeutic dose. This may result in the concentration or volume of the injection being increased, the destabilization of colloids, the aggregation of particles or a prolonged infusion time. Additionally, patients may be exposed to significant quantities of excipients whose long-term biological effects and toxicity are not yet fully understood [133,134]. Nanoparticles should also be on demand released: sustained steady-state sustained or rapid burst release to balance bioavailability and efficacy [135]. Meanwhile, the behavior of nanoparticles injected intravenously in physiological fluids is different from that in aqueous suspensions. Blood components will interact with nanoparticles to form a canopy, affecting their colloidal stability and leading to changes in pharmacokinetic behavior [136], and nanopreparations need to be stable after manufacture, storage and clinical administration.

#### 8.1.2. Biological Factors

There are also significant challenges in the activation, metabolism and in vitro simulation and evaluation of nano-agents in vivo. Tumor pathophysiology has to be associated with the heterogeneity of human malignant tumors from the onset of nanoformulation development in order to overcome biological barriers, accomplish tumor targeting and minimize uptake by non-target organs. Insufficient knowledge on patient disease heterogeneity and pharmacokinetic behavior of nanoformulations is the main reason for poor efficacy of promising nanoformulations in clinical studies [137,138,139]. Nanoparticles with high specific surface area induce lung inflammation, oxidative stress and vary in distribution, retention and excretion in vivo due to differences in size/surface properties [140,141]. When endocytosis is the dominant uptake route, nanoparticles can persist in cells for extended periods of time and their long-term toxicity mechanism remains unknown. Systematic pharmacokinetic, pharmacodynamic and toxicological studies are needed urgently [142]. Therefore, preclinical data must systematically evaluate efficacy, safety, bioavailability and pharmacokinetics in a variety of animal models which mimic true pathological conditions. Furthermore, it is imperative to optimize in vivo performance, dosing regimens and treatment regimens for specific clinical diseases. In addition, it is crucial to understand the impact of cancer progression on nanotherapeutics in order to identify subgroups of patients who are more amenable to nanotherapeutics. Most nanosystems still depend on EPR effects to obtain tumor enrichment [143], however, tumors display significant inter- and intra-patient heterogeneity with disease progression, making it difficult to achieve therapeutic advantage with a one size fits all approach. Moreover, whilst ligand-targeting nanosystems can improve drug internalization in tumor regions, a corresponding increase in clinical efficacy is rarely observed. No consensus exists on the optimal ligand density and it depends on the characteristics of the molecular target [144,145]. In order to anticipate in vivo complications or toxicity, it is necessary to simultaneously know the activity/toxicity of the drug, its interaction with biological constituents and the effect of target release rate and off-target accumulation. Therefore, from excipient screening, process optimization to purification steps should be pragmatic design. Even when common excipients are known to be safe, the complexity of synthetic components, coatings and ligands can dramatically alter in vivo activity, bioavailability and toxicological profiles [146].

#### 8.1.3. Regulatory Factors

Although numerous nanotherapies have been marketed, regulatory ethics and policy continue to evolve worldwide, constraining the commercialization potential of nanotherapies [147]. Safety and efficacy regulations for conventional drugs are still applied when assessing nanomedicines; regulators must also take special care to consider the effect of their physical and chemical properties on their distribution in vivo and interactions with biofilms and tissues. As there is no universal standard for classifying nanotechnology products, the same product may be classified as a drug or a medical device in different countries, further complicating the approval pathway. In light of the growing number of polymer-based nano-preparations, there is an urgent need for the development of a regulatory framework to accommodate it. Each polymer system has a different dose, route of administration, frequency and indication that must be assessed on a case-by-case basis. For this purpose, nanoparticle-specific registration requirements should be developed to fill gaps in device and drug regulations pertaining to clinical trial design, patient recruitment, formulation complexity, mode of administration, pharmacokinetics, pharmacodynamics and safety. Over-regulation increases the cost of listing, so it is crucial to avoid over-approval whilst maintaining safety and quality. Regulatory agencies in many countries, particularly China, the US, Japan, the UK, the EU and other regulatory agencies currently differ significantly in their oversight of nano-preparations, with no formal guidelines for nanodrugs having been issued [148]. Thus, it is necessary to develop globally harmonized regulatory norms with key countries and collaborate to develop proprietary analytical techniques and unified toxicological testing and compliance standards for different nanomaterials and structures to ensure the efficacy and safety of nano-preparations.

### 8.2. Trends in Future Research

Looking back at the last 20 years of research, the following key points need to be focused on in the current CPT nanodrug delivery system research:

#### 8.2.1. Optimization for Nano-Delivery Systems

Targeted delivery of nanoparticles depends on the EPR effect, but its efficiency varies with tumor heterogeneity. Rapid and quantitative EPR imaging techniques are desperately needed to instruct nanocarrier design. Meanwhile, the synthesis technique of nano-delivery system also needs to be further optimized and surface modification should be investigated for precise delivery. Highly biocompatible and degradable materials for carriers should be explored. External stimulus localization, topical administration to circumvent biological barriers and triggered on-demand delivery should be explored. The development of multi-response stimulus-type linkers and cascade chemical bonding, combined with combination therapy strategies such as immunotherapy and light therapy, could further enhance the efficacy.

#### 8.2.2. The Application of Emerging Technologies

Artificial intelligence (AI) has a huge application prospect in the field of nanodrug delivery systems, including target selection, structure modeling, toxicity evaluation, drug release monitoring and so on [149,150]. Currently, the application of AI in the development of CPT nanodrugs is still in its early stage. To develop more accurate, GLP-compatible preclinical animal models; meanwhile, the organoid technology and fluorescence labeling technology can also be used for evaluating the toxicity of nanodrugs, monitoring distribution and release behavior, assisting drug screening and personalized treatment, and accelerating the clinical conversion of CPT nanodrugs.

#### 8.2.3. Multidisciplinary and Industrial Collaboration

Although nanoparticles have been used as drug carriers, they still have issues such as long-term stability, large-scale production and high cost. Multidisciplinary collaboration and industrialization should be enhanced to address the issues of large-scale production, packaging and cost of nanodrug delivery systems and facilitate their clinical use. Standardize academic research practices; identify and validate biomarkers of efficacy/toxicity. It is anticipated that standardization, deepening of tumor biology and use of biomarkers will improve the success rate of new CPT nano-formulations entering the clinic.

## 9. Conclusions

In general, as multidisciplinary technology advances, the range of CPT compounds for nanodrug delivery systems continues to grow. Currently, various targeted delivery systems have been developed with their own merits and limitations, offering efficient carriers for tumor therapeutic drugs accurate delivery, enhancing the solubility and bioavailability of CPT, mitigating toxic and side effects, combining with PTT, magnetic therapy, immunotherapy, gene therapy, etc. to enhance the efficacy of tumor treatment. Although most of them are still in the research stage, CPT nanodrugs have demonstrated remarkable superiority over traditional drugs. The successful marketing of some therapeutic nanomedicines and the achievement of positive clinical outcomes has given developers hope. However, CPT nanomedicines still encounter challenges in stability, bioavailability and safety in clinical applications, and further research and clinical trials are required to validate and optimize nanomedicine formulations based on clinical outcomes. With continuous research and development in multidisciplinary technologies, we anticipate that these innovative CPT nanodrugs will undergo clinical transformation in the near future, becoming potent weapons in the fight against tumor diseases.

## Figures and Tables

**Table 1:** Pharmacological properties and clinical development overview of camptothecin and its derivatives.

Compound Name	Hydrophobicity and Physicochemical Properties	Primary Antitumor Indications	Main Toxicities/Side Effects	Development Stage
CPT	Strongly hydrophobic, extremely poor water solubility, E-ring lactone prone to hydrolytic ring-opening and inactivation	Broad-spectrum solid tumors (lung, liver, gastric, colorectal cancer, etc.)	Main adverse reactions include myelosuppression, gastrointestinal discomfort, diarrhea, hepatonephrotoxicity, alopecia, fatigue and cholinergic syndrome with varying severity	Not used clinically
HCPT	Hydrophobic, slightly improved water solubility and stability vs. CPT	Lung, liver, gastric, bladder cancer; leukemia		Clinically used (China)
9-MeO-CPT	Moderately hydrophobic, significantly enhanced lipophilicity	Lung, pancreatic, breast cancer		Preclinical/basic research
7-Et-CPT	Enhanced lipophilicity and structural stability, increased hydrophobicity	Colorectal, lung cancer		Preclinical/basic research
SN-38	Moderately hydrophobic, moderate water solubility, extremely high in vitro antitumor activity	Breast, gastric, urothelial cancer		Preclinical/basic research
CPT-11	Highly water-soluble prodrug, efficiently activated in vivo	Metastatic colorectal cancer, small cell lung cancer, cervical cancer		Globally approved
TPT	Good water solubility, significantly improved lactone stability vs. CPT	Small cell lung cancer, ovarian cancer, cervical cancer, neuroblastoma		Globally approved
9-NC	Strongly lipophilic, excellent oral absorption, poor water solubility	Lung, pancreatic, ovarian cancer, leukemia		Clinical stage (partial approval)
Belotecan	Good water solubility, strong tumor tissue penetration	Small cell lung cancer, ovarian cancer		Approved anticancer drug (South Korea)

**Table 2:** Summary of in vivo and in vitro antitumor experiments of CPT-class compound nanomedicine delivery systems.

Nanostructure Type	Therapy Modality	Cell Line	Tumor Model	In Vitro/In Vivo	References
HCPT liposome	Chemotherapy		Hepatoma H22 and Sarcoma S180 subcutaneous tumors in mice	In vivo	[21]
HCPT liposome	Chemotherapy and Sonodynamic Therapy	4T1-luc	Metastatic lung cancer model in mice constructed with 4T1-luc cells	In vitro/In vivo	[22]
mPEG-HCPT NPs	Chemotherapy				[24]
Poly(malic acid)-HCPT conjugate	Chemotherapy	SW480		In vitro	[25]
Micelles self-assembled from SN-38 encapsulated by mPEG-PLA	Chemotherapy	HCT116, BEL-7402	HCT116 cell subcutaneous tumor in nude mice	In vitro/In vivo	[27]
Camptothecin prodrug-mPEG-PLA micelles	Chemotherapy	CT26		In vitro	[28]
HCPT and siMCT-4 loaded SiO_2_ NPs	Chemotherapy and Immunotherapy	RAW 264.7, B16F10, 4T1	Subcutaneous tumors induced by B16F10 or 4T1 cells in Balb/c mice	In vitro/In vivo	[30]
HCPT-loaded Au NPs	Chemotherapy, Photothermal Therapy	KB, 4T1	4T1 cell subcutaneous tumor in BALB/c mice	In vitro/In vivo	[31]
HCPT-loaded Au NPs	Chemotherapy	MDA-MB-231	Subcutaneous tumors induced by MDA-MB-231 cells in nude mice	In vitro/In vivo	[32]
CPT NPs functionalized with PVA-coated MWCNTs and GO NPs	Chemotherapy	MDA-MB-231		In vitro	[33]
HCPT loaded human serum albumin NPs	Chemotherapy	HepG2	Subcutaneous tumors induced by HepG2 cells in nude mice	In vitro/In vivo	[36]
HCPT loaded glycyrrhetinic acid-bovine serum albumin NPs	Chemotherapy	SMMC7721		In vitro	[37]
CPT nanocrystals drug coated with hyaluronic acid	Chemotherapy	MDA-MB-23, MCF-7, HepG2		In vitro	[40]
HCPT and doxorubicin (DOX) self-assembled composite NPs	Synergistic Chemotherapy	MDA-MB-231, MCF-7, T47D		In vitro	[41]
HCPT nanosuspension	Chemotherapy		Subcutaneous tumors induced by H22 sarcoma cells in ICR mice	In vivo	[42]
Tetrahedral framework self-assembled from CPT modified with ethyl bromide and DNA modified with phosphorothioate	Chemotherapy	HCT116, MCF-7	HCT116 cell subcutaneous tumor in nude mice	In vitro/In vivo	[44]
Cys-DNA and CPT assembled into nanospheres	Chemotherapy				[45]
Hf-TP-SN nMOF with an X-ray triggerable SN38 prodrug	Synergistic Radiotherapy and Chemotherapy	CT26, 4T1, MC38 cells	CT26 and 4T1 cell tumors in BALB/c mice	In vitro/In vivo	[46]
CPT@mCOF@DOX-lipid	Synergistic Chemotherapy	4T1 cells	4T1 tumor model was created by subcutaneously injecting 4T1 cells into the upper hindlimbs of female BALB/c mice	In vitro/In vivo	[47]
RGD targeting peptides and CPT molecules conjugated PDC drug	Chemotherapy	MCF-7, MDA-MB-231, EJ	Orthotopic MCF-7 breast cancer tumor-bearing nude mouse model, EJ bladder cancer subcutaneous tumor-bearing mouse model	In vitro/In vivo	[51]
CPT-conjugated PSMA-responsive pentapeptide prodrug NPs	Chemotherapy	LNCaP-FGC, HepG2, MCF-7, HeLa, DU145, PC-3	Subcutaneous tumors induced by MCF-7 cells in BALB/c nude mice	In vitro/In vivo	[52]
Anti-Trop-2 antibody and SN-38 conjugated antibody-drug (ADC)	Chemotherapy	MDA-MB-468, BxPC-3, NCI-N87	Subcutaneous xenograft tumors respectively induced by BxPC-3 pancreatic cancer cells and NCI-N87 gastric cancer cells in female NCr nude mice	In vitro/In vivo	[53]
CPT loaded macroporous carbon carrier NPs	Chemotherapy				[59]
Folic acid-capped polyrotaxane-camptothecin conjugated NPs	Chemotherapy	A2780		In vitro	[60]
CPT-TK-FUDR (CTF) and HA-modified IR780 (HAIR)self-assemble to form an attractive nano-bomb (HAIR/CTF NPs)	Chemotherapy, Photothermal and Photodynamic Therapy	4T1	Subcutaneous tumors induced by 4T1 cells in Balb/c mice	In vitro/In vivo	[61]
SN38 loaded PDA-ALN NPs	Chemotherapy, Photothermal Therapy	NIH3T3, MSCs, MDA-MB-231, PC-9	Orthotopic tibial tumors induced by MDA-MB-231-Luc cells in BALB/c nude mice	In vitro/In vivo	[62]
CPT and epirubicin (EPI) co-loaded into a novel ferritin (ins-FDC) drug	Chemotherapy	U87MG, HepG2, MCF7-MDR, hASMC	Intracranial glioma model induced by U87MG-LUC cells, Lung metastasis liver cancer model induced by HepG2-LUC cells, Drug-resistant breast tumor model induced by MCF7-MDR cells	In vitro/In vivo	[64]
HCPT-loaded menthol and casein assembled NPs	Chemotherapy	C6	Orthotopic glioma model induced by C6 cells in nude mice	In vitro/In vivo	[65]
CPT nanocrystals coated with 4T1 cell membranes and loaded with the photosensitizer ICG	Chemotherapy, Photothermal Therapy	4T1, HeLa, HUVEC, J774A.1	Subcutaneous tumors induced by 4T1 cells in BALB/c mice	In vitro/In vivo	[68]
HCPT-loaded hepatocellular carcinoma cell membrane-coated liposomes	Chemotherapy	HepG2		In vitro	[69]
CPT-loaded exosomes	Chemotherapy, Radiotherapy		Subcutaneous xenograft tumors of patient-derived cervical cancer tissue in BALB/c nude mice	In vivo	[70]
CPT heterodimer prodrug self-assembled CSSP NPs	Chemotherapy	4T1, 3T3	4T1 cells were injected subcutaneously into the right back of female BALB/c mice	In vitro/In vivo	[71]
Aptamer/cell-penetrating peptide-camptothecin prodrug, i.e., Apt/CPP-CPTD NPs	Chemotherapy	Miapaca cells	Miapaca orthotopic pancreatic cancer xenograft models were established using luci Miapaca cells(Nude mice)	In vitro/In vivo	[73]
Self-targeted polymersomal co-formulation of doxorubicin, camptothecin and FOXM1 aptamer	Synergistic Chemotherapy	A549 and SK-MES-1 cells	SK-MES-1 cells were transplanted subcutaneously into the right flank of C57BL/6 nude mice	In vitro/In vivo	[74]
CPT-loaded MOFMIL-53(Fe) nanoparticles functionalized with polydopamine (PDA)	Chemotherapy	MCF-7	Injection of MCF-7 cells into the yolk sac of zebrafish embryos	In vitro/In vivo	[79]
PDA nanoparticles loaded with CPT-conjugated polymer prodrug	Chemotherapy, Photothermal therapy	HepG2, HeLa	Subcutaneous tumors induced by HeLa cells in BALB/c nude mice	In vitro/In vivo	[80]
ICG-encapsulated CPT prodrug copolymer micelles	Chemotherapy, Photodynamic Therapy	HeLa, H22	Subcutaneous tumors induced by H22 cells in BALB/c mice	In vitro/In vivo	[83]
A nanoassembly composed of FF-based photosensitive derivatives (PPA-DA) and hypoxia-activated camptothecin prodrugs (N-CPT)	Chemotherapy, Photodynamic Therapy	4T1 cells	The subcutaneous breast cancer xenograft bearing 4T1 cells female BALB/c mice	In vitro/In vivo	[84]
CPT prodrug nanogel	Chemotherapy	Hep G2	Subcutaneous Hep G2 cell tumors in BALB/c nude mice	In vitro/In vivo	[87]
Polymer nanoparticles loaded with HCPT and methotrexate (MTX)	Synergistic Chemotherapy	HepG2, Bel-7402		In vitro	[88]
Spherical nanovesicles composed of SN38 linked to short-chain OEG	Chemotherapy	MCF-7, KB, HT-29, SKOV-3	BCap37 and SKOV-3 cells were implanted into the peritoneal cavity of mice separately	In vitro/In vivo	[89]
MMP-2 peptide, CPT, and all-trans retinoic acid self-assembled NPs	Chemotherapy	NCI-H460, A549	Subcutaneous tumors induced by NCI-H460 cells in mice	In vitro/In vivo	[92]
Drug-peptide conjugate Asp-Thr-Lys-Thr-Gly-Pro-Ala-Lys(SA CPT)-Phe-Phe-Nap (1-CPT-Nap)	Chemotherapy	L929, MIA PaCa-2 cells	Female BALB/c nude mice bearing MIA PaCa-2 xenograft tumors	In vitro/In vivo	[93]
DOX and CPT co-loaded mesoporous CoFe2O4@PDA@ZIF-8 sandwich nanocomposite	Chemotherapy, Photothermal Therapy	Hep G2	Subcutaneous tumors induced by HepG2 cells in nude mice	In vitro/In vivo	[101]
Tumor-homing peptide-functionalized NPs loaded with 10-HCPT	Chemotherapy	MDA-MB-231	Subcutaneous tumors induced by MDA-MB-231 cells in nude mice	In vitro/In vivo	[105]
CPT-loaded polymer	Chemotherapy	Hela		In vitro	[106]
CPT-tR-PEG2000@BaTiO_3_ NPs	Chemotherapy	Panc02	Subcutaneous tumors induced by Panc02 cells in C57BL/6 mice	In vitro/In vivo	[107]
CPT and iron oxide (Fe_3_O_4_) loaded lipid-polymer NPs	Chemotherapy	MMTV-c-Neu Tg, Mouse Mammary Tumor Cell MT2		In vitro	[109]
CPT loaded SPION-based nanocomposites	Chemotherapy, Magnetothermal Therapy	Jurkat cells (E6)		In vitro	[110]
camptothecin (CPT)-based N-oxide prodrug	Chemotherapy, Radiotherapy	CT26 and HCT116 cell lines.	HCT116 tumor bearing female BALB/c nude mice	In vitro/In vivo	[111]
The block copolymer polyprodrug micelles loading verteporfin and CPT	X-Ray-Induced Photodynamic Therapy, Radiochemotherapy	HeLa cells	H22 cells were injected into the BALB/c female mice subcutaneously.	In vitro/In vivo	[112]
Janus tBT(tetragonal BaTiO_3_)@PDA-CPT NPs	Pyroelectric Dynamic Therapy, Chemotherapy and Photothermal Therapy	H22 and NIH3T3 cells	H22 cells was subcutaneously administered into each Kunming mouse	In vitro/In vivo	[113]
7-Ethyl CPT conjugated with RGD159 peptide nanodrug	Chemotherapy	NCI-H292, LoVo, 786-O	Subcutaneous PDX models of non-small cell lung cancer, colorectal cancer, renal cancer, and triple-negative breast cancer in NMRI nu/nu nude mice or NOG mice	In vitro/In vivo	[118]

## Data Availability

No new data were created or analyzed in this study. Data sharing is not applicable to this article.

## Abbreviations

The following abbreviations are used in this manuscript:

ADC	Antibody–Drug Conjugate
AI	Artificial Intelligence
ALN	Alendronate
ApDC	Aptamer–Drug Conjugate
ApoBD	Apoptotic Body
Bis	N,N-Methylenebisacrylamide
BSA	Bovine Serum Albumin
CA	Casein
CD44	Cluster of Differentiation 44
CNTs	Carbon Nanotubes
COF	Covalent Organic Framework
CPT	Camptothecin
CPT-11	Irinotecan
Cys-DNA	Cysteine-Modified DNA
Cy5	Cyanine 5
DOX	Doxorubicin
DPPC	1,2-Dipalmitoyl-sn-Glycerol-3-Phosphocholine
DPPG	L-α-Dipalmitoylphosphatidyl Glycerol
EPI	Epirubicin
EPR	Enhanced Permeability and Retention
FA	Folic Acid
FAP-α	Fibroblast Activation Protein Alpha
FA-PR-CPT	Folate-terminated Polyrotaxane-camptothecin
Fe_3_O_4_	Ferroferric Oxide
FR	Folate Receptor
FUDR	Floxuridine (5-fluorouracil deoxyribonucleoside)
GBI-10	Tenascin-C Targeting Aptamer
GO	Graphene Oxide
GSH	Glutathione
HA	Hyaluronic Acid
HCPT	10-Hydroxycamptothecin
Hf	Hafnium
HIF-1	Hypoxia-Inducible Factor-1
HSA	Human Serum Albumin
ICD	Immunogenic Cell Death
ICG	Indocyanine Green
IMMU-132	Sacituzumab Govitecan
IR780	Near-Infrared Dye
LIFU	Low-Intensity Focused Ultrasound
MAA	Methacrylic Acid
mCOF	Methoxylated Covalent Organic Framework
MDR	Multidrug Resistance
MMP	Matrix Metalloproteinase
MOFs	Metal–Organic Frameworks
mPEG	Methoxy Polyethylene Glycol
mPEG-PLA	Methoxy Polyethylene Glycol-Polylactic Acid
MRI	Magnetic Resonance Imaging
MTX	Methotrexate
MWCNTs	Multi-Walled Carbon Nanotubes
NIR	Near-Infrared
NK012	SN-38 Polymeric Micelle
NMR	Nuclear Magnetic Resonance
NP	Nanoparticle
N-oxide	Nitrone Oxide (functional group)
OEG	Oligoethylene Glycol
PCPT	Polymeric Camptothecin Prodrug
PDAC	Pancreatic Ductal Adenocarcinoma
PDA	Polydopamine
PDC	Peptide Drug Conjugate
PEDT	Pyroelectric Dynamic Therapy
PEG	Polyethylene Glycol
PEMA	2-(Pentamethyleneimino)Ethyl Methacrylate
PLA	Polylactic Acid
PLGA	Poly Lactic-co-Glycolic Acid
PMEA	Poly(2-Methacryloyloxyethyl Acrylate)
PPA-DA	Pheophorbide-Diphenylalanine Peptide
PS	Phosphorothioate
PSMA	Prostate-Specific Membrane Antigen
PTT	Photothermal Therapy
PVA	Polyvinyl Alcohol
RES	Reticuloendothelial System
RF	Radio Frequency
RGD	Arginine–Glycine–Aspartic Acid
RNA	Ribonucleic Acid
ROS	Reactive Oxygen Species
SDT	Sonodynamic Therapy
SEM	Scanning Electron Microscopy
siMCT-4	Small Interfering RNA targeting MCT-4
SiO_2_	Silicon Dioxide
SN-38	7-Ethyl-10-Hydroxycamptothecin
SPION	Superparamagnetic Iron Oxide Nanoparticle
tBT	Tetragonal Barium Titanate
TEM	Transmission Electron Microscopy
TK	Thioketal
TNBC	Triple-Negative Breast Cancer
TPP	Triphenylphosphine
TPT	Topotecan
Trop-2	Trophoblast Cell-Surface Antigen 2
UPLC-UV	Ultra-Performance Liquid Chromatography–Ultraviolet
VP	Verteporfin
ZIF-8	Zeolitic Imidazolate Framework-8
5-ALA	5-Aminolevulinic Acid
7-Et-CPT	7-Ethylcamptothecin
9-MeO-CPT	9-Methoxycamptothecin
9-NC	9-Nitrocamptothecin
